# Rps5-Rps16 communication is essential for efficient translation initiation in yeast *S. cerevisiae*

**DOI:** 10.1093/nar/gku550

**Published:** 2014-06-21

**Authors:** Arnab Ghosh, Supriya Jindal, Amber A. Bentley, Alan G. Hinnebusch, Anton A. Komar

**Affiliations:** 1 Center for Gene Regulation in Health and Disease, Department of Biological, Geological and Environmental Sciences, Cleveland State University, Cleveland, OH 44115, USA; 2 Laboratory of Gene Regulation and Development, Eunice K. Shriver National Institute of Child Health and Human Development, National Institutes of Health, Bethesda, MD 20892, USA

## Abstract

Conserved ribosomal proteins frequently harbor additional segments in eukaryotes not found in bacteria, which could facilitate eukaryotic-specific reactions in the initiation phase of protein synthesis. Here we provide evidence showing that truncation of the N-terminal domain (NTD) of yeast Rps5 (absent in bacterial ortholog S7) impairs translation initiation, cell growth and induction of *GCN4* mRNA translation in a manner suggesting incomplete assembly of 48S preinitiation complexes (PICs) at upstream AUG codons in *GCN4* mRNA. Rps5 mutations evoke accumulation of factors on native 40S subunits normally released on conversion of 48S PICs to 80S initiation complexes (ICs) and this abnormality and related phenotypes are mitigated by the *SUI5* variant of eIF5. Remarkably, similar effects are observed by substitution of Lys45 in the Rps5-NTD, involved in contact with Rps16, and by eliminating the last two residues of the C-terminal tail (CTT) of Rps16, believed to contact initiator tRNA base-paired to AUG in the P site. We propose that Rps5-NTD-Rps16-NTD interaction modulates Rps16-CTT association with Met-tRNA_i_^Met^ to promote a functional 48S PIC.

## INTRODUCTION

Eukaryotic translation initiation is a complex process involving multiple steps (1). For the majority of cellular mRNAs it starts with the recruitment of Met-tRNA_i_^Met^ to the 40S ribosomal subunit by eukaryotic-specific initiation factor eIF2 (1). In cooperation with initiation factors eIF3, 1/1A, the eIF2·GTP·Met-tRNA_i_^Met^ ternary complex (TC), binds the 40S ribosomal subunit yielding the 43S preinitiation complex (PIC) (1). The 43S complex then binds to the 5′-end of mRNA and scans in search of the initiation codon to form the 48S PIC. Following recognition of the start codon and eIF5-induced irreversible hydrolysis of eIF2-bound Guanosine-5'-triphosphate (GTP), eIF5B promotes joining of the 40S and 60S subunits and the elongation process begins (1).

While recent studies have yielded detailed insights into the mechanism of translation initiation, many details of the process remain unknown. The exact placement and orientation of initiation factors on the ribosomal surface, structural rearrangements accompanying various steps of initiation, the role played by ribosomal proteins, the timing (and kinetics) of factor association and release and, finally, the exact architecture of the 43S and 48S PICs are either unknown or just beginning to emerge (2,3).

X-ray structures of the yeast *Saccharomyces cerevisiae* 80S ribosome (4,5) are opening up new opportunities to investigate the mechanism and regulation of translation initiation in eukaryotic cells. Eukaryotic ribosomes have evolved to be structurally more complex than those of prokaryotes and it is believed that this complexity is directly related to the evolution of the translation apparatus, appearance of new translation factors as well as appearance of multiple sophisticated translation control mechanisms, absent in prokaryotic cells (1–6). One of the key features differentiating eukaryotic (yeast) from prokaryotic ribosomes is the extent of protein–protein interactions on the ribosome surface (4,6); however, the significance of these interactions is unknown.

We aim to understand the evolutionary complexity of the eukaryotic (yeast) ribosome by studying the structure and function of yeast ribosomal protein S5, which belongs to the rpS7 ribosomal protein family that includes rpS7 in bacteria and Rps5 in eukaryotes (4,7,8). The protein forms part of the exit (E) site, is essential for cellular viability and recent data suggest that yeast Rps5 functions in translation initiation (9,10) as well as 40S head formation (11). Rps5/S7 proteins possess conserved central and C-terminal regions and exhibit variability at the N-terminus, with fungi and fruit flies exhibiting the longest N-terminal tail regions as compared to bacteria and metazoans.

Here we provide a detailed functional analysis of the N-terminal domain (NTD) of yeast Rps5 suggesting that it communicates with Rps16 to influence events surrounding recruitment of TC and assembly of functional 48S PICs. Our biochemical analysis suggests that truncation of the Rps5 N-terminal region, or mutation of an NTD residue (K45) that contacts Rps16, compromises hydrolysis of eIF2-bound GTP (in the 48S PIC), increasing accumulation of eIF1 and eIF5B (in addition to the eIF2 accumulation noted before (10)) while reducing association of eIF5, thereby delaying subunit joining and progression of the 80S ribosome to the elongation-competent state. Remarkably, similar effects were observed on eliminating the last two residues of the C-terminal tail (CTT) of Rps16, believed to contact initiator tRNA when base-paired to AUG in the P site (2,3,12). These defects can be rescued by introducing an eIF5 mutant (G31R), reported previously to possess elevated GTPase-activating-protein (GAP) function (13). We therefore hypothesize that communication of Rps5 with Rps16 has evolved to enhance recruitment of the eukaryotic-specific eIF2·GTP·Met-tRNA_i_^Met^ ternary complex and regulated hydrolysis of eIF2-bound GTP, in a manner involving an altered location of the Rps16 CTT in the 40S decoding center.

## MATERIALS AND METHODS

### Yeast strains and growth methods

*rps5-Δ0*, *rps5-Δ1-13*, *rps5-Δ1-24*, *rps5-Δ1-30* and *rps5-Δ1-46* strains (Table 1) have been previously described (10), in which the chromosomal *RPS5* gene is deleted and replaced with a *kanMX* cassette and mutant or wild type (WT) *RPS5* alleles are present on high-copy plasmids and expressed from the strong *S. cerevisiae TEF1* promoter. The following strains of a similar design, *rps5-K41A Mat***a** *his3-1, leu2-0, ura3-0*, *rps5::kanMX*, <*yrps5-K41A*; *LEU2, 2μ*>, *rps5-F43G Mat***a** *his3-1, leu2-0, ura3-0*, *rps5::kanMX*, <*yrps5-F43G*; *LEU2, 2μ*>, *rps5-K45A Mat***a** *his3-1, leu2-0, ura3-0*, *rps5::kanMX*, <*yrps5-K45A*; *LEU2, 2μ*>, expressing yeast rps5 variants with the indicated amino acid substitutions, were obtained as follows: point mutations were first introduced into the *RPS5* gene in pTEF_yS5 plasmid (10) using side-directed mutagenesis and the following primers 5′-CAAACCGAGATTGCGTTGTTCAAC-3′ forward and 5′-GTTGAACAACGCAATCTCGGTTTG-3′ reverse (pTEF_yS5-K41A); 5′-GAGATTAAGTTGGGCAACAAATGGTC-3′ forward and 5′-GACCATTTGT TGCCCAACTTAATCTC-3′ reverse (pTEF_yS5-F43G); and 5′-AGTTGTTCAACGCATGGTCTTTTG-3′ forward and 5′-CAAAAGACCATGCGTTGAACAACT-3′ reverse (pTEF_yS5-K45A). The resultant pTEF_yS5 plasmids carrying mutant *RPS5* were transformed into the heterozygous diploid BY4743 *[4741/4742] MAT***a***/MATα, his3-1/his3-1, leu2-0/leu2-0, lys2-0/+, met15-0/+, ura3-0/ura3-0, RPS5/rps5::kanMX*. Transformants were allowed to sporulate using standard protocols (14) and tetrads were dissected. Haploid clones able to grow on YPED medium, resistant to G418 sulfate and expressing mutant *RPS5* were selected. The *rps5::kanMX* genotype was further verified by PCR using 5′-CAGGTGCGACAATCTATCG-3′ and 5′-GAAACGTTACGTTTAGAGACAATG-3′ primers.

**Table 1.** Strains of *S. cerevisiae*

Strain	Genotype	Source/reference
*rp5+ gcn2Δ*	*Mat*a *his3-1, leu2-0, ura3-0, rps5::kanMX, <yrps5; LEU2, 2μ> gcn2::hisG*	This work
*rps5-Δ0*	*Mat*a *his3-1, leu2-0, ura3-0, rps5::kanMX, <yrps5; LEU2, 2μ>*	(10)
*rps5-Δ1-13*	*Mat*a *his3-1, leu2-0, ura3-0, rps5::kanMX, <yrps5-13; LEU2, 2μ>*	(10)
*rps5-Δ1-24*	*Mat*a *his3-1, leu2-0, ura3-0, rps5::kanMX, <yrps5-24; LEU2, 2μ>*	(10)
*rps5-Δ1-30*	*Mat*a *his3-1, leu2-0, ura3-0, rps5::kanMX, <yrps5-30; LEU2, 2μ>*	(10)
*rps5-Δ1-46*	*Mat*a *his3-1, leu2-0, ura3-0, rps5::kanMX, <yrps5-46; LEU2, 2μ>*	(10)
*rps5-K41A*	*Mat*a *his3-1, leu2-0, ura3-0, rps5::kanMX, <yrps5-K41A; LEU2, 2μ>*	This work
*rps5-F43G*	*Mat*a *his3-1, leu2-0, ura3-0, rps5::kanMX, <yrps5-F43G; LEU2, 2μ>*	This work
*rps5-K45A*	*Mat*a *his3-1, leu2-0, ura3-0, rps5::kanMX, <yrps5-K45A; LEU2, 2μ>*	This work
*rps16+gcn2Δ*	*his3-1, leu2-0, met15-0, LYS, ura3-0, rps16B::kanMX4, rps16A::HIS3, <pGAL-RPS16A; LEU2, ARS1, CEN4> <pRPS28-RPS16; MET15, 2μ> gcn2::hisG*	This work
*rps16 WT*	*his3-1, leu2-0, met15-0, LYS, ura3-0, rps16B::kanMX4, rps16A::HIS3 <pGAL-RPS16A; LEU2, ARS1, CEN4> <pRPS28-RPS16A; URA3, 2μ>* or *his3-1, leu2-0, met15-0, LYS, ura3-0, rps16B::kanMX4, rps16A::HIS3 <pGAL-RPS16A; LEU2, ARS1, CEN4> <pRPS28-RPS16A; MET15, 2μ>*	This work
*rps16-R143G*	*his3-1, leu2-0, met15-0, LYS, ura3-0, rps16B::kanMX4, rps16A::HIS3 <pGAL-RPS16A; LEU2, ARS1, CEN4> <pRPS28-RPS16A; URA3, 2μ>*	This work
*rps16-RΔ*	*his3-1, leu2-0, met15-0, LYS, ura3-0, rps16B::kanMX4, rps16A::HIS3 <pGAL-RPS16A; LEU2, ARS1, CEN4> <pRPS28-RPS16A-R143Δ; URA3, 2μ>* or *his3-1, leu2-0, met15-0, LYS, ura3-0, rps16B::kanMX4, rps16A::HIS3 <pGAL-RPS16A; LEU2, ARS1, CEN4> <pRPS28-RPS16A-R143Δ; MET15, 2μ>*	This work
*rps16-YRΔΔ*	*his3-1, leu2-0, met15-0, LYS, ura3-0, rps16B::kanMX4, rps16A::HIS3 <pGAL-RPS16A; LEU2, ARS1, CEN4> <pRPS28-RPS16A-Y142ΔR143Δ; URA3, 2μ>* or *his3-1, leu2-0, met15-0, LYS, ura3-0, rps16B::kanMX4, rps16A::HIS3 <pGAL-RPS16A; LEU2, ARS1, CEN4> <pRPS28-RPS16A-Y142ΔR143Δ; MET15, 2μ>*	This work
*rps16-F46A*	*his3-1, leu2-0, met15-0, LYS, ura3-0, rps16B::kanMX4, rps16A::HIS3 <pGAL-RPS16A; LEU2, ARS1, CEN4> <pRPS28-RPS16A-F46A; MET15, 2μ>*	This work
*rps16-Y49G*	*his3-1, leu2-0, met15-0, LYS, ura3-0, rps16B::kanMX4, rps16A::HIS3 <pGAL-RPS16A; LEU2, ARS1, CEN4> <pRPS28-RPS16A-Y49G; MET15, 2μ>*	This work

*RPS16-WT*, *rps16-R143G* and *rpS16-YRΔΔ* strains were obtained as follows: the desired mutations were introduced into the *RPS16A* gene (expressed from *RPS28* promoter) in high-copy plasmid K1005 (Yeplac195-pRPS28-FLAG-RPS16; URA3; 2μ) (a kind gift from Dr Philipp Milkereit, University of Regensberg, Germany) by site targeted mutagenesis using primers 5′-CCAAAAATCTTACGGTTAAGAAATTGTGG-3′ forward and 5′- CCACAATTTCTTAACCGTAAGATTTTTGG-3′ reverse (*rps16-R143G*); 5′-GATTCCAAAAATCTTAAGAAATTGTGG-3′ forward and 5′-CCACAATTTCTTAAGATTTTTGGAATC-3′ reverse (*rpS16-YRΔΔ*). Plasmids containing desired mutations were then transformed into strain Y-318 (pGAL-RPS16A) *his3-1, leu2-0, met15-0, LYS, ura3-0*, *rps16B::kanMX4, rps16A::HIS3 <pGAL-RPS16A; LEU2, ARS1, CEN4*>, lacking the chromosomal genes encoding the two isoforms of Rps16 and harboring a low-copy plasmid containing *RPS16A* under the glucose-repressible *GAL* promoter (15) (a kind gift from Dr Philipp Milkereit, University of Regensberg, Germany). The resulting strains were grown in glucose containing media to block the expression from *pGAL-RPS16A; LEU2, ARS1, CEN4* and thus Rps16 expressed from Yeplac195-pRPS28-FLAG-RPS16 becomes the sole source of Rps16 protein expressed in these strains under glucose growth conditions. *RPS16*, *rps16-RΔ* and *rps16-YRΔΔ* yeast strains for *GCN4-LacZ* and *SUI5* assays were obtained as follows: K1005 vector (Yeplac195-pRPS28-FLAG-RPS16; URA3; 2μ) harboring RPS16 wild-type or mutants was digested with PstI/NarI and the pRPS28-FLAG-RPS16 was inserted into PstI/ClaI digested pRS421 (2μ, *MET15*) vector. The resulting pRS421_RPS16 constructs were transformed into Y-318 strains already harboring K1005 plasmid with RPS16 wild type sequence. K1005 constructs were eliminated from the resulting strains by 5-FOA selection, thus obtaining yeast strains expressing wild-type or mutant Rps16 (expressed from pRS421 plasmids). Yeast cultures were grown as indicated using either synthetic media containing 0.67% Difco yeast nitrogen base, 1% ammonium sulfate, 2% glucose (or galactose) and supplemented with the appropriate amino acids or YEPD medium (14). Transformation was done using the lithium acetate method (16). For polysome analysis, yeast cells were grown in YEPD medium with 2% glucose.

### Reporter plasmids

Yeast p180, p196, p227, p226, p209 and pM226 reporter plasmids (17,18) derivatives of YCp50 (*CEN*, URA3) vector bearing *GCN4-lacZ* alleles were kindly provided by Drs Thomas Dever and Alan Hinnebusch (National Institutes of Health). pRS SUI3-S264Y-U plasmid (derivative of pRS316 (*CEN*, URA3)) harboring *SUI3-S264Y* allele and YCp SUI5-G31R-U plasmid (derivative of YCplac33 (*CEN*, URA3)) harboring *TIF5-G31R* allele (19) were kind gifts of Dr Leoš Valášek (Institute of Microbiology, Academy of Sciences of the Czech Republic). *HIS4-LacZ* reporter constructs with AUG or UUG initiation codons (p367 and p391, respectively) for assaying Sui phenotypes (20) were kind gifts of Dr Alan Hinnebusch (National Institutes of Health). Plasmids were transformed into wt and RPS5 or RPS16 mutant strains and grown on the minimal YNB medium. *rps5+gcn2Δ* and *rps16+gcn2Δ* strains were generated by transforming ancestor strains (rps5 and rps16, respectively) with an XbaI/EcoRI fragment derived from construct B2806 (18) containing the disruption cassette 5′*-gcn2-hisG-URA3-hisG-gcn2-*3′. Ura^+^ colonies were picked and were counter-selected on 5-FOA plates. The *gcn2* deletion was confirmed by the strain's inability to grow under starvation conditions in the presence of sulfometuron methyl (SM) (an inhibitor of isoleucine-valine biosynthesis) and/or 3-amino-1,2,4-triazole (3-AT) (an inhibitor of histidine biosynthesis).

### Fractionation of polyribosomes

Fractionation of polyribosomes was done essentially as described before (9,10) using 10–50% (17 000 rpm., 18 h) and/or 10–30% (20 000 rpm., 18 h) sucrose gradients and a Beckman SW32.1 rotor. All procedures were performed at 4°C. Yeast cells from 50 ml of log phase culture were pelleted, treated for 10 min with 100 μg/ml cycloheximide and repelleted. Cell extracts were made by glass bead cell disruption (3–5 cycles of 1 min each), with intermittent cooling on ice. The following buffer was used: 100 mM KCl, 2.5 mM magnesium acetate, 20 mM HEPES·KOH, pH 7.4, 14.4 mM β-mercaptoethanol, 100 μg/ml cycloheximide. Cell debris was removed by centrifugation at 7000 rpm for 8 min and polyribosomes were resolved by sucrose density gradient centrifugation as indicated. Gradients were collected using the ISCO Programmable Density Gradient System with continuous monitoring at 254 nm using an ISCO UA-6 absorbance detector. Analysis of ratios of 80S monosomes to polyribosomes was done essentially as before (10). Fractionation of cell extracts using formaldehyde cross-linking was done as described by Nielsen *et al*. (18). For western blotting, proteins collected from sucrose gradient fractions were solubilized in sodium dodecyl sulfate (SDS) polyacrylamide sample buffer for 10 min at 95°C, chilled on ice for 5 min and loaded onto polyacrylamide gel.

### Western blotting

Western blotting was done following standard procedures (21). Anti-rpS5 antibodies derived against the AIKKKDELERVAKSNRC C-terminally conserved rpS5 peptide has been described previously (10). The anti-eIF2α (22), anti-eIF5B (23), anti-eIF1 (24) and anti-eIF5 antibodies were kindly provided by Drs Thomas Dever and Alan Hinnebusch (National Institutes of Health). Goat anti-rabbit HRP-conjugated antibodies and enhanced chemiluminescence detection kit (ECL™, GE Healthcare, Piscataway, NJ, USA) were used for detection.

### β-Galactosidase assays

For *GCN4-lacZ* assays, cells were grown in a minimal synthetic (SD) medium supplemented with appropriate amino acids containing 2% galactose (for 2 h). To invoke amino acid starvation, sulfometuron methyl (SM) (final concentration 1 μg/ml), or 3-amino-triazole (3-AT) (final concentration 30 mM) was then added and the incubation was continued for additional 5 h. Cells were harvested, and extracts were prepared by subsequent cycles of cell freezing in liquid nitrogen and thawing at 37°C. For assaying Sui phenotypes, cells were grown in minimal synthetic media in presence of 2% galactose as inducer and β-galactosidase activity was assayed using the whole cell extract. β-Galactosidase activity was measured following the protocol described in Clontech Yeast Protocols Handbook using *O*-nitrophenyl β-d-galactopyranoside as a substrate.

### Miscellaneous

Molecular cloning was performed following standard procedures. Deoxyribonucleic acid (DNA) sequencing was accomplished by the DNA Sequencing Core facility at Cleveland Clinic. Sodium dodecyl sulfate polyacrylamide gel electrophoresis was performed according to Laemmli (25). Yeast genomic DNA was isolated using the DNA-PureTM Yeast Genomic Kit (PureBiotech, Middlesex, NJ, USA) following the manufacturer's protocol.

## RESULTS

Biochemical analysis of mutant yeast strains carrying truncated Rps5 variants suggested that the NTD of yeast Rps5 impacts the ability of 40S ribosomal subunits to function properly in translation initiation (10). Specifically, we reported increased eIF2 association with the mutant 40S subunits containing Rps5 lacking 30 or 46 N-terminal amino acid residues (in strains *rps5-Δ1-30* and *rps5-Δ1-46*, respectively) compared to 40S subunits in the WT strain (10). We thus proposed that the N-terminal truncation of Rps5 either affects recruitment of eIF2-ternary complexes to 40S subunits or influences ribosomal association of eIF2 by modulating eIF5-stimulated hydrolysis of eIF2-bound GTP and/or subsequent dissociation of the eIF2·GDP complex (10).

To gain further insights into the exact step in the initiation process affected by Rps5-NTD truncations, we embarked on a more detailed analysis of translation initiation in Rps5 mutant cells by taking advantage of *GCN4* translational control (26). Regulation of *GCN4* translation is exerted via a reinitiation process involving four small upstream open reading frames (uORFs) preceding the *GCN4* ORF and is known to be a sensitive indicator of translation initiation defects *in vivo* (for a review, see (26)). Following translation of the 5′-proximal uORF (uORF1) reinitiation depends on *de novo* recruitment of the eIF2 TC, which is required to recognize the next AUG codon, and is thus exquisitely sensitive to the eIF2·GTP level (for review, see (26)). Amino acid starvation derepresses *GCN4* translation by provoking reduced availability of the TC (for review, see (26)).

### Translation reinitiation is compromised in Rps5 mutants

**Figure 1.**
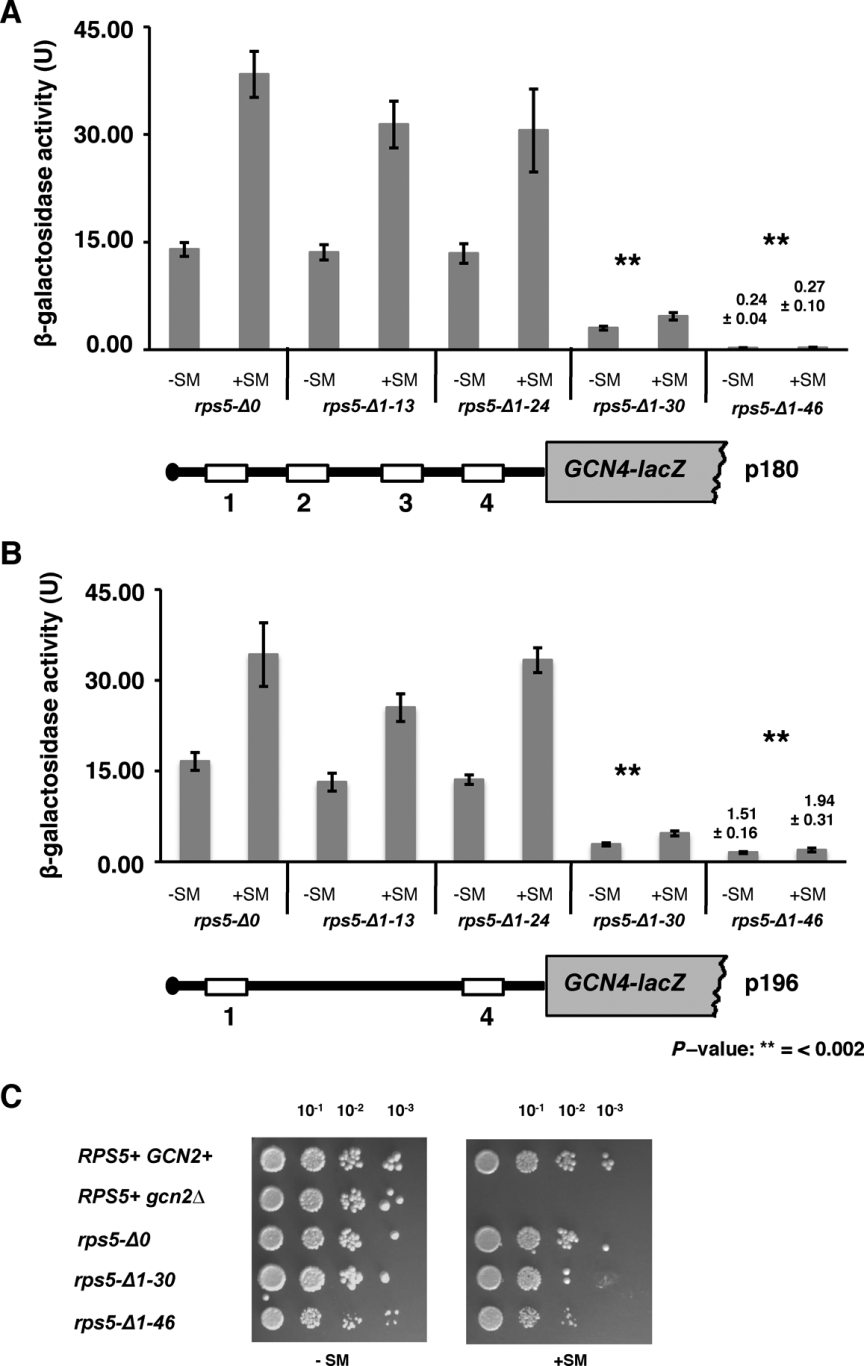
Rps5 N-terminal region is essential for reinitiation in yeast cells. (A, B) Expression of reporter *GCN4-lacZ* constructs. (**A**) p180 containing wild type *GCN4* mRNA leader (four uORFs) and (**B**) p196 containing only uORFs 1 and 4, were transformed into wild type *rps5-Δ0* and isogenic *rps5-Δ1-13, rps5-Δ1-24, rps5-Δ1-30, rps5-Δ1-46* yeast strains. β-Galactosidase activity (units) are shown; measured under normal and amino acids starved conditions (+SM). (**C**) Yeast cell growth. Serial dilutions of strains spotted onto minimal media under non-starved (−SM), or amino acid (aa) starved conditions (+SM), respectively.

**Figure 2.**
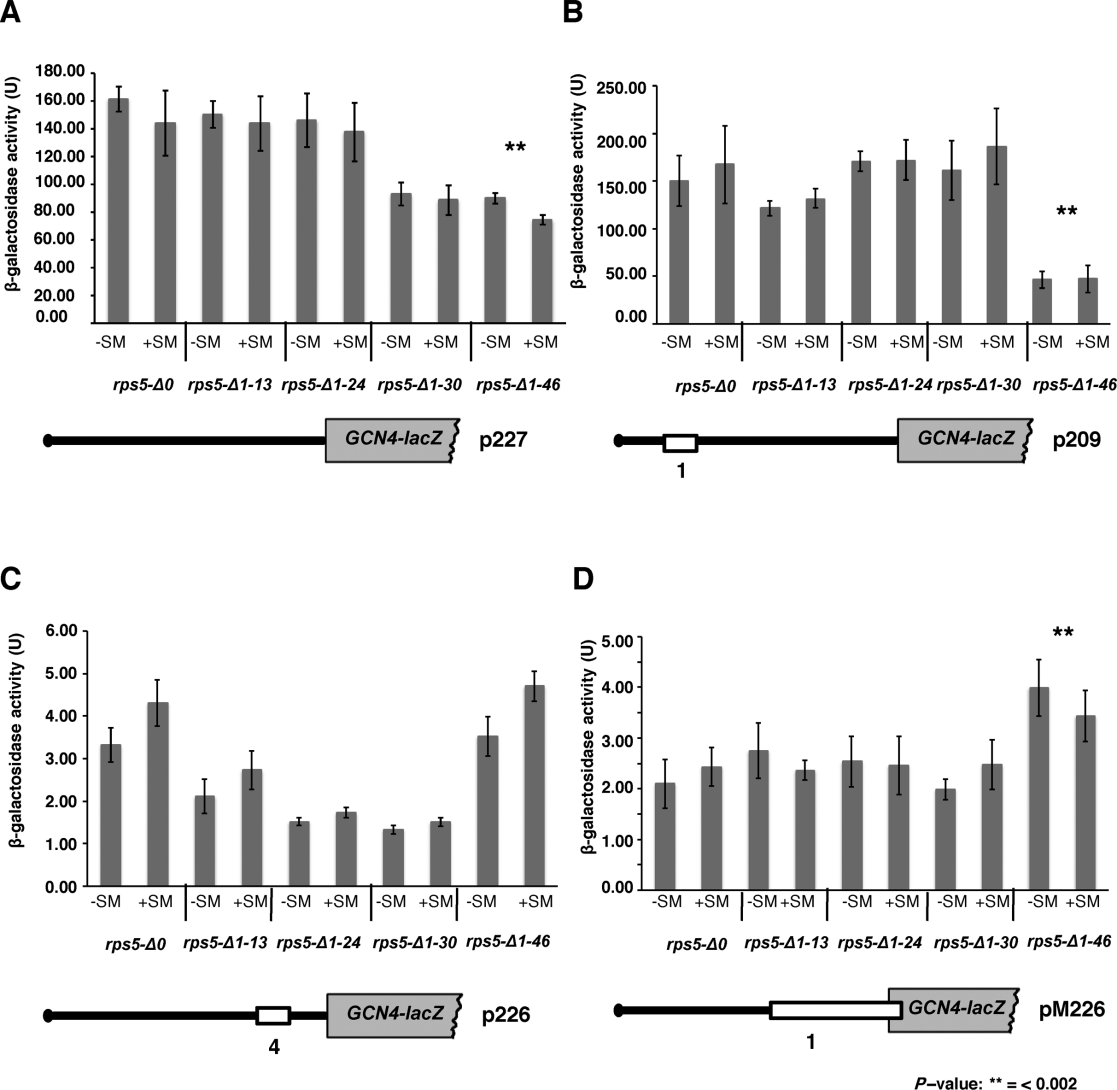
Truncation of Rps5 46 N-terminal amino acid residues confers leaky scanning phenotype. Expression of reporter *GCN4-lacZ* constructs. *rps5-Δ0* and isogenic *rps5-Δ1-13, rps5-Δ1-24, rps5-Δ1-30, rps5-Δ1-46* strains were transformed with (**A**) p227 containing uORFs less *GCN4* mRNA leader; (**B**) p209 containing uORF1; (**C**) p226 containing only uORF4 and (**D**) pM226 containing uORF1 extended into *GCN4* ORF. β-Galactosidase activity (units) are shown; measured under normal (−SM) and aa starved conditions (+SM) as in Figure 1.

To assess reinitiation in yeast strains expressing N-terminally truncated versions of Rps5, we assayed a set of *GCN4-lacZ* reporters with different arrangements of the uORFs (Figures 1 and 2 and Supplementary S1A). Plasmids were transformed into wild type strain *rps5-Δ0* and its isogenic derivatives *rps5-Δ1-13*, *rps5-Δ1-24*, *rps5-Δ1-30* and *rps5-Δ1-46* carrying different truncated *RPS5* genes. Cells were treated with sulfometuron methyl (SM), which inhibits isoleucine-valine biosynthesis, to derepress *GCN4* expression (for review, see (26)). We found that truncation of 13 or 24 N-terminal amino acids in Rps5 does not substantially affect reinitiation in constructs containing all four uORFs (reporter plasmid p180, Figure 1A) or only uORFs 1 and 4 (p196, Figure 1B), as strains *rps5-Δ1-13* and *rps5-Δ1-24* exhibit basal levels of β-galactosidase activity under non-starvation conditions (−SM), and derepressed levels of activity under amino acid starvation conditions (+SM), that are nearly identical to those seen in the WT strain. Upstream ORFs 2 and 3 are known to be functionally redundant with uORF4 such that uORF1 and uORF4 are sufficient for nearly WT regulation of reinitiation on *GCN4* mRNA (17). By contrast, *GCN4-lacZ* expression from p180 was reduced by ∼4-fold in *rps5-Δ1-30* and by ∼40-fold in *rps5-Δ1-46* in comparison with the WT strain (Figure 1A). Thus, while *rps5-Δ1-30* retains a low-level induction of reinitiation (∼2-fold), both the basal level of reinitiation and its induction were almost completely abrogated in *rps5-Δ1-46*. The same holds true for the p196 construct that contains only uORFs 1 and 4 (Figure 1B). The *GCN4-lacZ* expression data were corroborated by growth assays (Figure 1C). Growth of *rps5-Δ1-46* was compromised under amino acid starved conditions (Figure 1C, +SM), suggesting that GCN4 fails to efficiently activate the expression of genes encoding amino acid biosynthetic enzymes in this mutant (26). These observations led us to conclude that the *rps5-Δ1-46* mutant exhibits a strong General control non-derepressible (Gcn^−^) phenotype (26).

We have previously found (using a reporter plasmid carrying *lacZ* with a short 5′UTR (∼50 nt) under control of the *GAL1* promoter) that cap-dependent initiation was reduced in the *rps5-Δ1-30* and *rps5-Δ1-46* strains by ∼30% and ∼50%, respectively (10). We observed a similar reduction (∼2-fold) in cap-dependent initiation levels for a *GCN4-lacZ* reporter construct (p227) devoid of all four upstream open reading frames (uORFs) and thus possessing a long 5′UTR of ∼600 nt (Figure 2A and Supplementary S1A). This suggests that, independently of the 5′UTR length, initiation in *rps5-Δ1-46* is reduced by only about 2-fold, while reinitiation is almost completely blocked. This finding led us to conduct a more thorough analysis of the role played by the Rps5 NTD in translation reinitiation.

### The rps5-*Δ*1*-*46 mutant displays translation initiation defects downstream of 48S complex formation including a leaky scanning phenotype

The Gcn^−^ phenotype observed in the *rps5-Δ1-46* strain could arise for a number of reasons. Firstly, it is possible that the mutant ribosomal subunits are unable to resume scanning after they translate uORF1 and thus dissociate from the mRNA after terminating translation at the uORF1 stop codon. It is known that only those ribosomes that can successfully translate uORF1, terminate and resume scanning downstream, can bypass uORFs 2, 3, 4 and reinitiate translation at the *GCN4* ORF (26). To test whether mutant ribosomes can resume scanning after translating uORF1, we assayed a *GCN4-lacZ* reporter (on p209) containing uORF1 at its original position (350 nt from the *GCN4* AUG codon) as the only uORF in the leader (17) (Figure 2B and Supplementary Figure S1A). The uORF1 possesses specific surrounding sequences, both 5′ and 3′, which allow a large fraction of ribosomes to remain attached to the mRNA, resume scanning, and reinitiate downstream (26). Thus, the absence of uORFs 2–4, and the equivalent frequencies of reinitiation at *GCN4* following uORF1 translation that occur in non-starvation and starvation conditions, confers high-level, constitutive expression from p209 (Figure 2B and Supplementary Figure S1A, *rps5-Δ0*, +SM versus –SM). A failure to resume scanning following uORF1 translation would reduce expression of the p209 construct. As expression of this construct in the *rps5-Δ1-13*, *rps5-Δ1-24* and *rps5-Δ1-30* strains were comparable to that in *rps5-Δ0* (Figure 2B and Supplementary Figure S1A), it appears that none of these Rps5 truncations affect the ability of the mutant 40S ribosomal subunits to resume scanning after translating uORF1. This was not the case for the *rps5-Δ1-46* mutant in which expression of p209 was reduced by a factor of ∼3 (Figure 2B and Supplementary Figure S1A). It is important to note, however, that the level of p209 expression in *rps5-Δ1-46* remains more than 100-fold higher than that observed for the p180 construct in this mutant (Figure 1A). By contrast, in the WT strain, expression of the p209 construct is only ∼4-fold higher than that from p180 under starvation conditions, indicating that in WT cells a substantial fraction (∼25%) of the 40S subunits that resume scanning following uORF1 translation bypass uORFs 2–4 and reinitiate at *GCN4* instead. The corresponding data for p209 and p180 in the *rps5-Δ1-46* mutant indicate that <1% of the 40S subunits scanning downstream from uORF1 bypass uORFs 2–4 and reinitiate at *GCN4*. Accordingly, the failure to induce *GCN4-lacZ* expression from p180 in this mutant does not stem primarily from the reduced ability of the mutant 40S ribosomal subunits to resume scanning after translating uORF1. In WT cells, expression from p209 is expected to be ∼50% of that observed for p227 (17), owing to the fact that only ∼50% of the ribosomes that translate uORF1 can resume scanning and reinitiate at the *GCN4* start codon (26). Because comparison of the results in Figure 2A and B for *rps5-*Δ*0* cells did not reveal this difference, we made a side-by-side comparison of expression from p227 and p209 in the *rps5-*Δ*0* strain and observed the predicted 2-fold higher expression for p227 versus p209 (data not shown). We presume that day to day variation in the measurement of β-galactosidase activities may have obscured this 2-fold difference in the results of Figure 2A and B.

We further considered a second possibility to account for the strong Gcn^−^ phenotype of the *rps5-Δ1-46* strain, wherein the mutant *rps5-Δ1-46* 40S subunits would exhibit a reduced rate of scanning that allows the TC to be acquired before the reinitiating ribosomal subunits bypass uORF4, thereby increasing reinitiation at uORF4 and producing an equivalent reduction in reinitiation at *GCN4.* To test this possibility, we assayed the p226 reporter containing a single uORF4 in its natural position (17). uORF4 is known to be the critical negative regulator of *GCN4* expression (26). Indeed, *GCN4-lacZ* expression from the p226 construct in WT cells is ∼5-fold lower compared to that from the p180 or p196 constructs in which the presence of uORF1 allows a fraction of reinitiating ribosomes to bypass the uORF4 start codon (compare data for strain *rps5-Δ0* in Figures 1A, B and 2C). Under normal circumstances, ribosomes that translate uORF4 reinitiate at the *GCN4* start codon at a very low frequency partly because of the low propensity for resumption of scanning by post-termination ribosomes at the uORF4 stop codon, but also because of its proximity to the *GCN4* AUG, as shown by the fact that *GCN4* expression from a solitary uORF4 construct was partially derepressed by increasing the distance (scanning time) separating uORF4 from *GCN4* (26,27). Thus, it can be predicted that increasing the time required to scan from the uORF4 stop codon to the *GCN4* AUG by decreasing the rate of scanning by mutant 40S subunits would increase *GCN4-lacZ* expression from the p226 construct (28). This was not observed in the *rps5-Δ1-46* mutant however, as expression of the p226 reporter was indistinguishable between this mutant and the WT strain (Figure 2C). Thus, it appears that a reduced rate of scanning by reinitiating 40S subunits does not contribute to the strong Gcn^−^ phenotype of the *rps5-Δ1-46* mutant. The other three Rps5 mutants display 2- to 3-fold reductions in expression of the p226 construct compared to the WT strain (Figure 2C and Supplementary Figure S1A), which might indicate that reinitiation following uORF4 translation is even less efficient in these mutants than in WT cells. Presumably, this defect is not seen for the p180 and p196 constructs (Figure 1A and B) because the great majority of *GCN4-lacZ* expression for these regulated constructs stems from reinitiating ribosomes that have bypassed uORF4.

We sought to confirm our conclusion that the *rps5-Δ1-46* mutation does not decrease the rate of scanning by examining constructs pM199 and pG67 in which the distance between solitary uORF1 and the *GCN4* AUG is smaller than normal (140 nt and 32 nt, respectively, Supplementary Figure S1B) versus the WT spacing of 350 nt present in construct p209 (Figure 2B). We observed a progressive decrease in β-galactosidase expression from these three constructs as the distance between uORF1 and *GCN4* AUG decreased in both WT and *rps5-Δ1-46* strains (compare expression levels from p209 in Figure 2B, Supplementary Figure S1A and pM199, pG67 in Supplementary Figure S1B for *rps5-Δ0* and *rps5-Δ1-46* strains). This reduction is expected from the progressive decrease in the amount of time available to reassemble an initiation complex by reinitiating 40S subunits before they reach the *GCN4* AUG codon, diminishing the frequency of reinitiation (31). Importantly, expression from the pM199 and pG67 constructs was lower in the *rps5-Δ1-46* mutant versus the *rps5-Δ0* WT strain (Supplementary Figure S1B), whereas a decreased rate of scanning in the mutant should evoke higher expression by increasing the probability of reinitiation at the *GCN4* AUG, especially for pG67 where the time required to scan the 32 nt uORF1-*GCN4* interval is normally so small. The decreased expression of pM199 and pG67 in the *rps5-Δ1-46* strain (Supplementary Figure S1B), also observed above for p209 (Figure 2B), supports our conclusion that the resumption of scanning following uORF1 translation is reduced in the mutant, decreasing the frequency of reinitiation at the *GCN4* AUG codon.

A third possible defect underlying a Gcn^−^ phenotype is the failure of scanning 40S subunits to recognize the uORF1 AUG codon, as translation of uORF1 is required to generate reinitiating 40S subunits that can bypass uORFs 2–4 and reinitiate at *GCN4* when TC levels are reduced by starvation. To investigate this, we assayed *GCN4-lacZ* expression from the solitary-uORF1 reporter on pM226 in which uORF1 is extended to overlap the *GCN4-lacZ* coding region (Figure 2D and Supplementary Figure S1A), which destroys the ability of ribosomes to reinitiate at *GCN4* after terminating at the elongated uORF1 stop codon. Hence, because initiation at uORF1 is normally so efficient and 40S subunits do not ‘leaky scan’ past the uORF1 AUG codon, this extension of uORF1 very effectively represses *GCN4-lacZ* expression (29) (compare in WT cells expression for pM226 in Figure 2D with p209 in Figure 2B). However, we observed only a slight ≤2-fold increase in *GCN4-lacZ* from pM226 in *rps5-Δ1-46* compared to *rps5-Δ0* (Figure 2D and Supplementary Figure S1A). While this degree of leaky scanning of the uORF1 AUG codon likely makes a small contribution to the strong reduction in expression of the p180/p196 constructs, it clearly cannot account for the virtually complete abrogation of *GCN4-lacZ* expression from these two regulated constructs (Figure 1A and B). Presumably, the small increase in leaky scanning of uAUG1 in the mutant is obscured for constructs p209, pM199 and pG67 by the decreased frequency of reinitiation following uORF1 translation deduced for these constructs.

Having eliminated defects in the resumption of scanning, rate of scanning, or leaky scanning of uORF1 as the origin of the strong Gcn^−^ phenotype in the *rps5-Δ1-46* strains, it seems most likely that the reinitiating mutant 40S subunits are unable to bypass uORFs 2–4 at low TC concentrations (caused by amino acid starvation) because they are forced to queue up behind a mutant 40S subunit stalled at the AUG codon of uORF 2, 3 or 4 that is unable to complete the assembly of a 48S PIC competent for subsequent steps of initiation following AUG recognition, such as completion of GTP hydrolysis by eIF2, release of eIF2·GDP or other eIFs from the 40S subunit, or subunit joining. This delay would allow sufficient time to rebind TC to the 40S subunits queued up behind it, which would then go on to reinitiate at the uORF AUG when it is eventually cleared by the stalled 40S subunit, preventing them from reinitiating at *GCN4*. In fact, our finding that the elimination of uORFs 2 and 3 in construct p196 increased *GCN4-lacZ* in comparison to the WT p180 construct under starvation conditions specifically in the *rps5-Δ1-46* mutant (Figure 1B and Supplementary Figure S2) supports this idea, as the absence of uAUGs 2 and 3 would eliminate two of the three sites where queuing would be imposed in the mutant. We note, however, that although we observed certain discrepancies in expression from p180 in *rps5-Δ1-46* mutant (Supplementary Figure S1A versus S2; starved conditions) potentially due to overall very low levels of β-galactosidase activity in this strain, we have always observed a statistically significant increase in expression from p196 construct as compared to p180 in this strain (Supplementary Figure S1A and S2). Consistent with these data, a relatively smaller increase in expression under starvation conditions was observed in the *rps5-Δ1-46* mutant on elimination of only uORF2 in construct p195 (Supplementary Figure S2, please compare 1.1 for p180, 1.64 for p195 and 2.01 for p196 at +SM). In addition, *GCN4-lacZ* expression from construct pG29, which lacks uORFs 2–3 as the result of a deletion between uORFs 1 and 4, also was elevated compared to the p180 construct in the *rps5-Δ1-46* mutant under starvation conditions, whereas expression from pG29 is lower than that given by p180 in starved WT (*rps5-Δ0*) cells (Supplementary Figure S2). The reduced pG29 expression seen in WT cells under starvation conditions, as well as the higher expression of this construct compared to p180 observed in nonstarved WT cells, both are consistent with previous results (29); which were attributed to increased bypass of uORF4 in nonstarvation conditions, coupled with increased bypass of the *GCN4* AUG in starvation conditions, by reinitiating 40S subunits. Our finding that expression from pG29 under starvation conditions is increased rather than decreased in the *rps5-Δ1-46* mutant (Supplementary Figure S2) supports the idea that queuing of reinitiating 40S subunits (diminished by deletion of uORFs 2–3 in pG29) makes an important contribution to the Gcn^−^ phenotype of the *rps5-Δ1-46* mutant. We presume that expression of pG29 under starvation conditions remains lower in the *rps5-Δ1-46* mutant versus WT cells (Supplementary Figure S2) because queuing of reinitiating subunits still occurs upstream of uORF4. Thus, our analysis of different *GCN4-lacZ* reporters leads us to predict that a step of initiation following AUG recognition by the scanning PIC is defective in *rps5-Δ1-46* cells.

### The rps5-Δ1-46 mutant exhibits altered association of eIF1, eIF5 and eIF5B with 48S complexes and does not exhibit a Sui^−^ phenotype

Dynamic interaction of initiation factors with 40S subunits is important for translational control of *GCN4* (for review, see (26)). In yeast, eIF1, the eIF2 TC, eIF3 and eIF5 comprise the multifactor complex (MFC), which may bind to the 40S ribosomal subunits as a preformed unit (30). Mutations that disrupt interactions of these initiation factors within the MFC or impair their recruitment to the ribosome affect reinitiation (for review, see (26)). We were thus interested to know whether the N-terminal Rps5 truncation evokes altered interactions of the mutant 40S subunits with MFC components, particularly eIF1 as this factor ensures accurate start codon recognition by blocking Pi release from the hydrolyzed eIF2·GDP·Pi complex and stabilizes the open, scanning-conducive conformation of the 40S subunit at non-AUG codons, while being released from the PIC on AUG recognition (for review, see (1,31)). Moreover, eIF1 is known to reside below the P-site and in the vicinity of the E-site (for review, see (1)) and hence in proximity to Rps5. Mutations in Rps5 might impede eIF1 release to decrease the efficiency of AUG recognition and confer the observed increase in leaky scanning of *GCN4* uAUG1, and the postulated failure to complete the initiation process at uORFs 2–4 (with attendant queuing of scanning ribosomes) in the *rps5-Δ1-46* mutant (Figure 2D and Supplementary Figure S1A).

**Figure 3.**
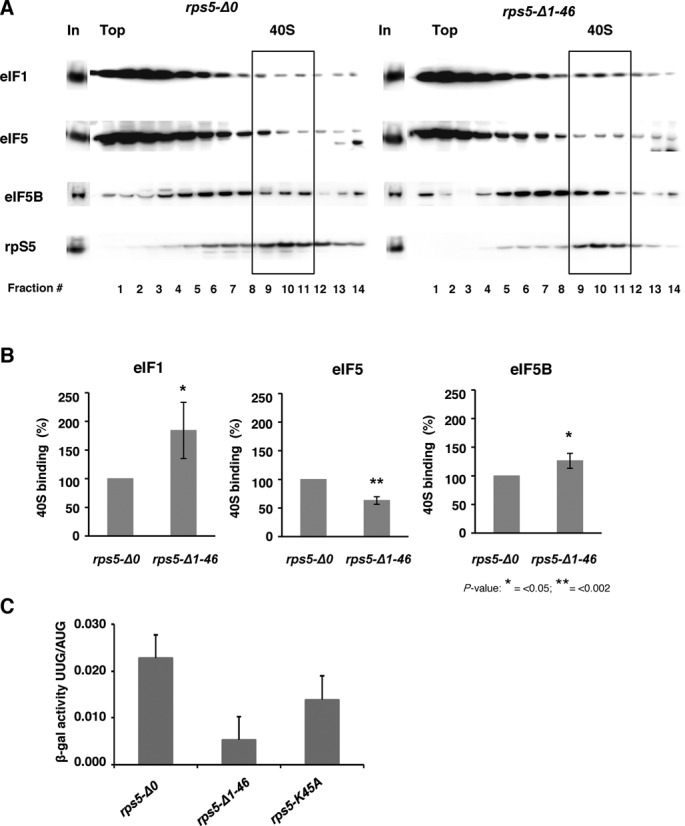
Association of eIF1, eIF5 and eIF5B with 40S ribosomal subunits in wt and mutant (*rps5-Δ1-46*) yeast strains and the stringency of AUG recognition on *HIS4* mRNA (Sui phenotype). (**A**) Extracts from isogenic wt (*rps5-Δ0*) and Rps5 mutant (*rps5-Δ1-46*) strains were resolved by sucrose density gradient (10–30%) sedimentation. Western blot analyses were done using antibodies against eIF1, eIF5 and eIF5B and the ribosomal protein S5, respectively. ‘In’ for input - represents a 7% portion of each gradient fraction. Analysis of eIF1, eIF5 and eIF5B association was done using whole cell extract cross-linking with formaldehyde. (**B**) Association of eIF1, 5 and 5B with the 40S was quantified and expressed as percentage of 40S binding normalized against Rps5. (**C**) *rps5-Δ0*, *rps5-Δ1-46* and *rps5-K45A* strains harboring reporter *HIS4-LacZ* constructs with either AUG or UUG initiation codons, respectively, were assayed for β-galactosidase activity. Mean ratio of expression from UUG to AUG reporter constructs are shown with standard errors from three experiments.

Accordingly, we assessed the association of eIF1 with native 40S subunits in extracts of WT and *rps5-Δ1-46* cells that were treated with formaldehyde to fix 43S/48S complexes *in vivo* and preserve their interactions with MFC components during separation of the whole cell extract (WCE) by sedimentation through sucrose density gradients. Western analysis of the gradient fractions with antibodies against eIF1 and the 40S subunit protein Rps5 revealed an increase in the eIF1:Rps5 ratio in the 40S fractions of the mutant versus WT cells (Figure 3A; top panel and 3B). We also checked the 40S-association of eIF5, as the eIF5 stimulates hydrolysis of eIF2-bound GTP and also plays an important role in eIF1 dissociation, Pi release and efficient AUG recognition through its interactions with eIF1, eIF2, eIF3 and eIF1A (for review, see (31)) (24,32). Moreover, there is evidence that eIF5 and eIF1 have antagonistic, positive and negative functions, respectively, in the efficiency and accuracy of start codon recognition ((33,34), reviewed in (35)). Western blotting of the gradient fractions revealed decreased association of eIF5 with the 43S/48S complexes formed in the *rps5-Δ1-46* mutant compared to the wild type strain (Figure 3A; top panel and B). We further decided to check the 40S-association of eIF5B, as this factor ensures efficient 40S and 60S subunit joining in the last stage of the initiation pathway (for review, see (1)) and, thus, it was possible that the Rps5 truncation impairs eIF5B recruitment and thereby compromises formation of elongation-competent 80S ribosomes. We found that association of the mutant 40S ribosomal subunits with eIF5B was not reduced, and was even increased compared to its association with WT 40S subunits (Figure 3A; bottom panel and B).

It is worth recalling here that we previously reported an accumulation of eIF2 in the 43S/48S complexes in the *rps5-Δ1-46* mutant as compared to the WT strain (10). Taking these data together with the results in Figure 3AB and our analysis of *GCN4* translational control, leads us to suggest that the initiation defect in the *rps5-Δ1-46* mutant involves events occurring following AUG recognition by the scanning PIC. One interesting possibility is that release of eIF1 is impeded, with an attendant delay in both Pi release from eIF2·GDP·Pi and subsequent dissociation of eIF2·GDP from the 48S complex (36), which in turn delays the completion of subunit joining by 48S-associated eIF5B. In view of recent evidence that eIF1 and eIF5 compete for one or more binding sites on the 40S subunit (24,32), the accumulation of eIF1 in the PIC might be coupled to the apparent decrease in eIF5 association shown in Figure 3A for the *rps5-Δ1-46* mutant.

Yeast eIF1 was originally identified by the isolation of mutations that reduce the stringency of AUG recognition on *HIS4* mRNA (Sui^−^ phenotype) (37). Sui^−^ mutants increase initiation at the UUG codon encoding the third amino acid in the His4 protein, thus bypassing the requirement for the AUG start codon in *HIS4* mRNA for yeast growth on medium lacking histidine (37). Extensive analysis of Sui^−^ mutations in eIF1 has established that they elevate aberrant initiation at UUG codons by reducing the recruitment of eIF1, or enabling its inappropriate release from the 48S PIC at non-AUG codons, owing to weakened interactions of eIF1 mutants with the 40S subunit or MFC components (reviewed in (31) (38,39). It was further suggested that gain-of-function Sui^−^ mutations in eIF2β or eIF5 that accelerate GTP hydrolysis by eIF2 and induce premature release of Met-tRNA_i_^Met^ from eIF2·GDP on the scanning 40S subunit enable ribosomes to initiate translation from the UUG codon in *HIS4* mRNA (13). We therefore also tested whether the *rps5-Δ1-46* mutant is defective in rejecting UUG start codons by assaying two matched *HIS4-lacZ* reporter constructs containing AUG or UUG at the first codon of the *HIS4* coding sequence and calculating the ratio of expression from the UUG versus AUG reporter. Interestingly, the *rps5-Δ1-46* mutant exhibits a significant reduction in the UUG:AUG initiation ratio compared to the WT strain (Figure 3C and Supplementary Figure S3). This hyperaccuracy phenotype is similar to that conferred by so-called *ssu* mutations, which suppress the elevated UUG:AUG initiation ratios conferred by *sui* mutations in the same or other initiation factors (reviewed in (31)). The suppression of the UUG:AUG initiation ratio in *rps5-Δ1-46* cells (Figure 3C and Supplementary Figure S3) would be compatible with a defect in start codon recognition resulting from a delay in eIF1 or Pi release if we assume that this defect has a relatively greater impact at UUG codons where mismatch with the anticodon of tRNA_i_ already disfavors these events. This interpretation is supported by the results shown in (Supplementary Figure S3) indicating that the *rps5-Δ1-46* mutation produces a greater reduction in expression of the *HIS4-lacZ* UUG reporter (∼10-fold) compared to that of the *HIS4-lacZ* AUG reporter (∼3-fold).

### A gain-of-function variant of eIF5 rescues the translational defects and slow growth phenotype of rps5-Δ1-46 strain

The results obtained using *GCN4-lacZ* constructs (Figures 1A,B, 2, and Supplementary Figure S1, S2) combined with the biochemical data revealing elevated accumulation of eIF2 (10), eIF1 and eIF5B, along with decreased association of eIF5 with 43/48S complexes (Figure 3A and B) suggested that there might be a delay in eIF1 dissociation or Pi release from eIF2-GDP after scanning 48S PICs encounter the AUG codons at the *GCN4* uORFs in *rps5-Δ1-46* mutant cells. As explained above, this delay in assembling elongation-competent 48S PICs at the *GCN4* uAUGs 2–4 would create a barrier for all reinitiating 40S subunits scanning the leader and increase the time available to re-acquire the TC before the uAUGs 2–4 are encountered, thereby restoring translation of the uORFs and reducing translation of *GCN4* for the Gcn^−^ phenotype. If this defect occurs during primary initiation events for most other mRNAs, it could also account for the Slg^−^ phenotype of the *rps5-Δ1-46* mutant. We reasoned that if this interpretation is correct, it might be possible to suppress both the Slg^−^ and Gcn^−^ phenotypes of *rps5-Δ1-46* by introducing into this mutant plasmid-borne *SUI3-S264Y* or *SUI5* mutant alleles encoding the gain-of-function Sui^−^ variants eIF2β-S264Y and eIF5-G31R, respectively, reported to elevate the intrinsic rate of GTP hydrolysis by eIF2 (*SUI3-S264Y*) or GAP function of eIF5 (*SUI5*) (13). Subsequent biochemical analysis of *SUI5* has revealed that it also stabilizes the closed conformation of the PIC, in which start codon recognition occurs, specifically at near-cognate start codons (including UUG), independent of any effect on rates of GTP hydrolysis (40).

**Figure 4.**
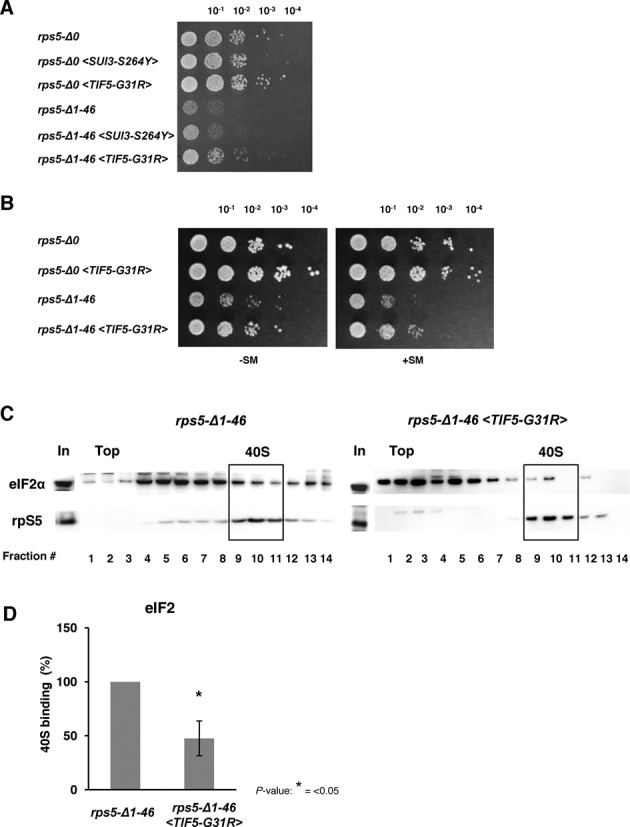
Increased GAP function of eIF5 rescues slow growth phenotype and initiation defects of *rps5-Δ1-46* strain. (**A**) Yeast cell growth. Yeast *rps5-Δ0* (Wt) and *rps5-Δ1-46* strains (in particular harboring *SUI3-S264Y* and *TIF5-G31R* alleles) were spotted onto YNB agar plates with 2% glucose. (**B**) *TIF5-G31R* allele complements the Gcn^−^ phenotype in *rps5-Δ1-46*. Serial dilutions of yeast strains were spotted onto YNB agar plates with 2% glucose and incubated under non-starved (−SM) conditions or aa starved conditions (+SM), respectively. (**C**) Association of initiation factor eIF2 with 40S subunits in *rps5-Δ1-46* and *rps5-Δ1-46* <*TIF5-G31R>* yeast strains. Western blot analysis of individual fractions with antibodies against eIF2α and rpS5 is shown. ‘In’ for input - represents a 7% portion of each gradient fraction. *TIF5-G31R* allele rescues enhanced accumulation of eIF2 on mutant 40S ribosomal subunits. (**D**) Association of eIF2 with the 40S subunits was quantified and expressed as percentage of 40S binding normalized against Rps5.

By comparing the growth rates of the WT strain and the *rps5-Δ1-46* mutant harboring these plasmids (Figure 4A), we found that *SUI5*, but not *SUI3-S264Y* diminished the Slg^−^ phenotype of the mutant strain. It is important to mention that the enhanced hydrolysis of eIF2-bound GTP conferred by the *SUI3-S264Y* product was observed in the absence of eIF5 (13) and hence the difference in the effects observed here for *SUI3-S264Y* and *SUI5* might stem from the different mechanisms involved in their phenotypes. We further found that the *SUI5* allele partially rescues the Gcn^−^ phenotype of the *rps5-Δ1-46* mutant, increasing growth under conditions of amino acid starvation (Figure 4B). These results led us also to address whether introduction of *SUI5* would reverse the accumulation of eIF2 in 43S/48S complexes shown previously to occur in *rps5-Δ1-46* (10). Indeed, we found that association of eIF2 with native 40S subunits in strain *rps5-Δ1-46* was reduced by the introduction of *SUI5* (Figure 4C and D). Together, these data support the possibility that the translation initiation defect in the *rps5-Δ1-46* mutant involves delayed hydrolysis of eIF2-bound GTP or Pi release from eIF2·GDP·Pi in 48S PICs after encountering the AUG start codon.

### Disruption of Rps5-Rps16 interaction via Rps5 N-terminus confers a slow growth phenotype

**Figure 5.**
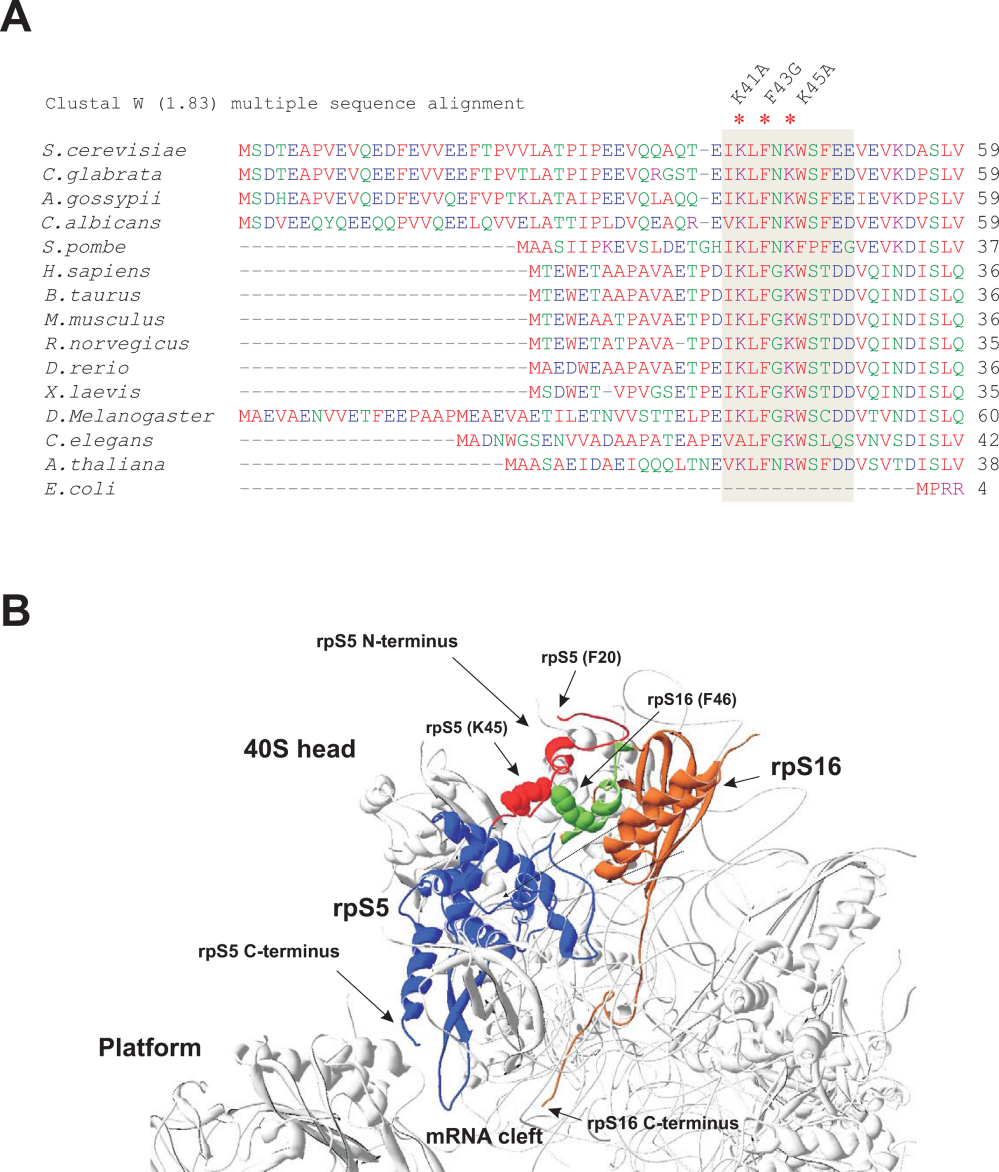
Sequence alignments of ribosomal protein S5/S7, the structure of yeast Rps5 and its ribosomal location relative to Rps16. (**A**) Sequence alignments of N-terminal amino acid regions of RpS5/S7 from various species. The amino acid residues, comprising residues 40–50 (in yeast Rps5) are boxed in gray. The three mutated residues are marked (*) and corresponding amino acid substitutions are shown. (**B**) Structures and ribosomal locations of Rps5 and Rps16. PDB files 3U5G and 3U5F were used and structures visualized using Swiss Pdbviewer (59). Rps5 is in blue with its eukaryote specific N-terminal extension in red. The Rps16 is shown in orange with part of it, which appears to interact with Rps5 N-terminus in green. Interacting Rps5 K45 (red) and F46 in Rps16 (green) are shown (van-der Waals radii of the side chain residues are shown). The view is from the solvent side of the 40S ribosomal subunit. The Rps5 N-terminus extends towards the solvent side and the Rps16 C-terminus is protruding towards the mRNA cleft.

We previously showed that eliminating all 50 N-terminal amino acids from Rps5 is lethal in yeast, and that global translation was compromised in *rps5-Δ1-30* and severely impaired in the *rps5-Δ1-46* mutant without a significant effect on ribosome biogenesis (10). These data, together with the results presented above, strongly suggest that the Rps5 N-terminal region (at least residues ∼40–50) is critical for translation and, hence, might have evolved in eukaryotes to perform an important function. Alignment of Rps5 from metazoans and fungi showed that this N-terminal region contains a number of highly conserved amino acids, including K41, F43 and K45 (Figure 5A). Interestingly, the crystal structure of the yeast 80S ribosome at 3.0 Å (3) revealed that the Rps5 N-terminal region interacts with the N-terminal region of Rps16 (belonging to the Rps9 family, which includes S9 in bacteria), and that conserved Rps5 residue K45 directly interacts with F46 in Rps16 (Figure 5B). Rps16 is located on the solvent side of the 40S head; however, it has a long protruding C-terminal tail (CTT) that reaches the mRNA cleft at the region occupied by the P-site tRNA (4,12). Thus, interaction of Rps5 and Rps16 via their respective N-terminal ends might affect the conformation of Rps16, including its CTT, and in turn perturb binding of TC to the PIC. It is also known that mammalian Rps5 and Rps16 mutually stabilize each other's binding to RNA and that the Rps5-Rps16 complex undergoes substantial structural rearrangement on RNA-binding (41).

**Figure 6.**
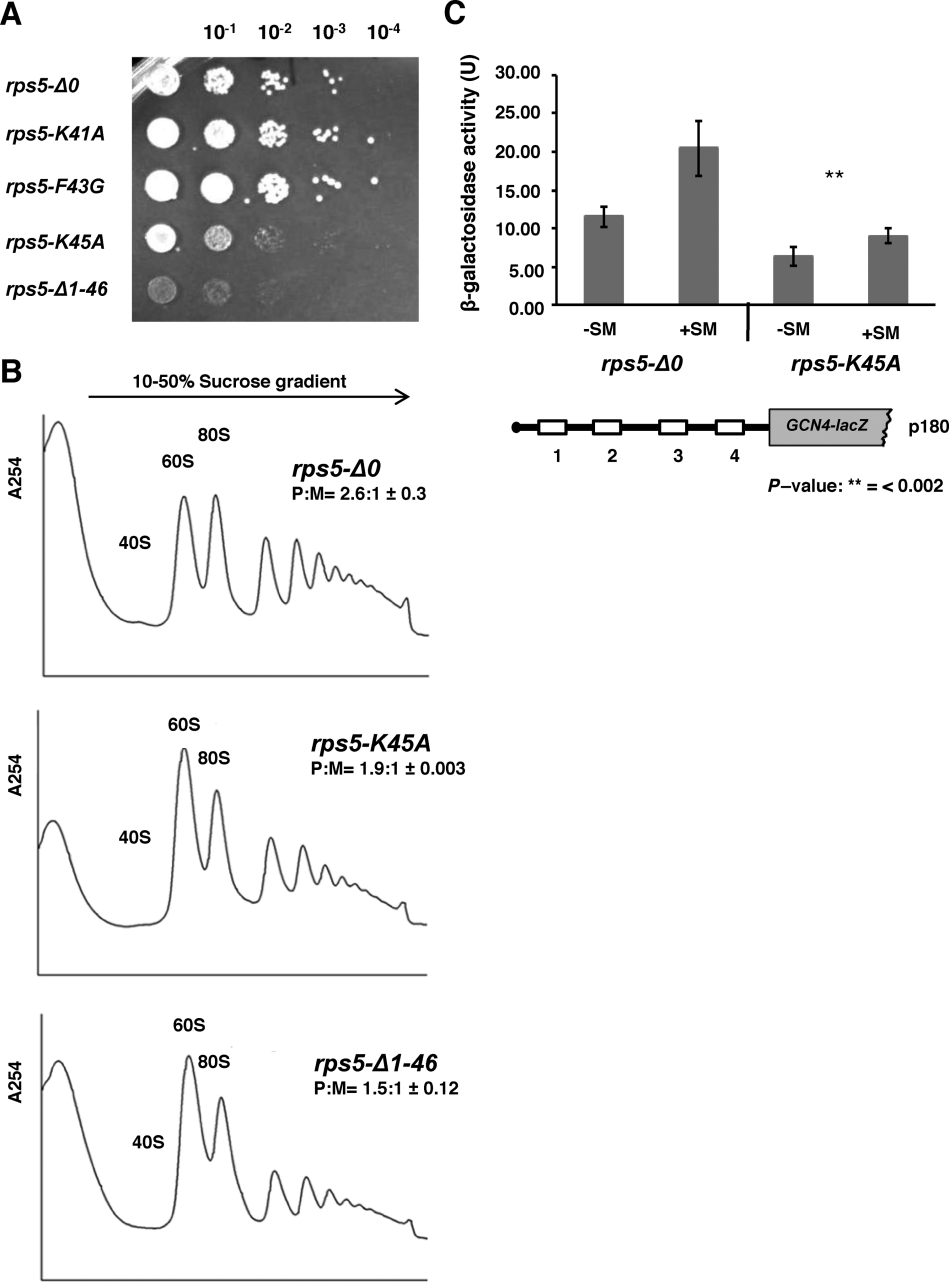
A single point K45A mutation in Rps5 confers phenotype as in *rps5-Δ1-46* strain. (**A**) Yeast cell growth. Serial dilutions of yeast strains with the indicated genotypes (harboring point mutation in Rps5; K41A, F43G and K45A) and *rps5-Δ1-46* were spotted onto YPD agar plates with 2% glucose. (**B**) Ribosome profiles of the wt, *rps5-Δ1-46* and *rps5-K45A* mutant yeast strains. Extracts were resolved in 10–50% sucrose density gradients. The ratios of the area under the polysomal (P) and 80S (M) peaks (P:M) are shown with ± standard errors. *rps5-Δ1-46* and *rps5-K45A* mutant yeast strains reveal very similar ribosome profiles. (**C**) *rps5-Δ0* and *rps5-K45A* yeast strains were transformed with reporter construct p180 containing wild type GCN4 mRNA leader (with all four uORFs) upstream of *GCN4-LacZ* fusion gene. The strains were assayed for β-galactosidase activity under normal, nutrient rich, (−SM) and aa starved (+SM) conditions as in Figure 1.

To test this possibility, we mutated conserved Rps5 residues K41, F43 and K45, substituting positively charged K41 and K45 with alanines and bulky aromatic F43 with glycine and obtained yeast strains in which the wild type yeast *RPS5* was replaced by the mutant *RPS5* alleles. Interestingly, *rps5-K41A* (harboring lysine to alanine substitution at Rps5 position 41) and *rps5-F43G* (harboring phenylalanine to glycine substitution at position 43) exhibited growth rates very similar to that of the wild type strain, while *rps5-K45A* displayed a pronounced Slg^−^ phenotype approaching that of the *rps5-Δ1-46* strain (Figure 6A). Analysis of polysomes by sedimentation of whole cell extracts through sucrose density-gradients further revealed a marked reduction in polysome (P) to monosome (80S) ratio (P:M) in the *rps5-K45A* mutant as compared to the wild type strain, albeit not as severe as that seen for the *rps5-Δ1-46* mutant (Figure 6B). A decrease in the P:M ratio is a characteristic phenotype of mutations that impair translation initiation without a commensurate reduction in the elongation or termination phases of translation, reducing the average number of ribosomes per mRNA. Importantly, we also found that the *rps5-K45A* mutant resembles the *rps5-Δ1-46* strain in displaying a Gcn^−^ phenotype, as indicated by increased sensitivity to SM (Supplementary Figure S4A) and reduced β-galactosidase activity expressed from the *GCN4-lacZ* reporter on p180 (Figure 6C and Supplementary Figure S4B); and also in displaying a reduced UUG:AUG ratio for the *HIS4-lacZ* reporters (Figure 3C and Supplementary Figure S3). Like the Slg^−^ phenotype and reduction in P:M ratio (Figure 6A and B), both defects were less pronounced in *rps5-K45A* versus the *rps5-Δ1-46* mutant. Interestingly, the reciprocal *F46A* mutation in *RPS16* confers a slow growth phenotype (Supplementary Figure S5A) similar in degree to that observed for the *rps5-K45A* mutant (Figure 6A). In addition, Rps16 Tyrosine 49 also forms contacts with the Rps5 N-terminus, and the *Y49G* mutation in *RPS16* (4,5) confers an even stronger Slg^−^ phenotype (Supplementary Figure S5A). Importantly, F46A mutation causes a similar reduction in polysome (P) to monosome (80S) ratio (P:M) (as in the *rps5-K45A* mutant) as compared to the wild *RPS16* type strain (Supplementary Figure S5B). Although further experiments are required to determine the exact consequences of these *RPS16* mutations, these data support the possibility that interaction between the N-terminal regions of Rps5 and Rps16 is critical for efficient translation initiation at a step following AUG recognition. This further raises the question of how structural changes on the solvent side of the 40S ribosomal subunit may affect TC recruitment and GTP hydrolysis.

### Rps16 C-terminal residues are critical for ribosome function

**Figure 7.**
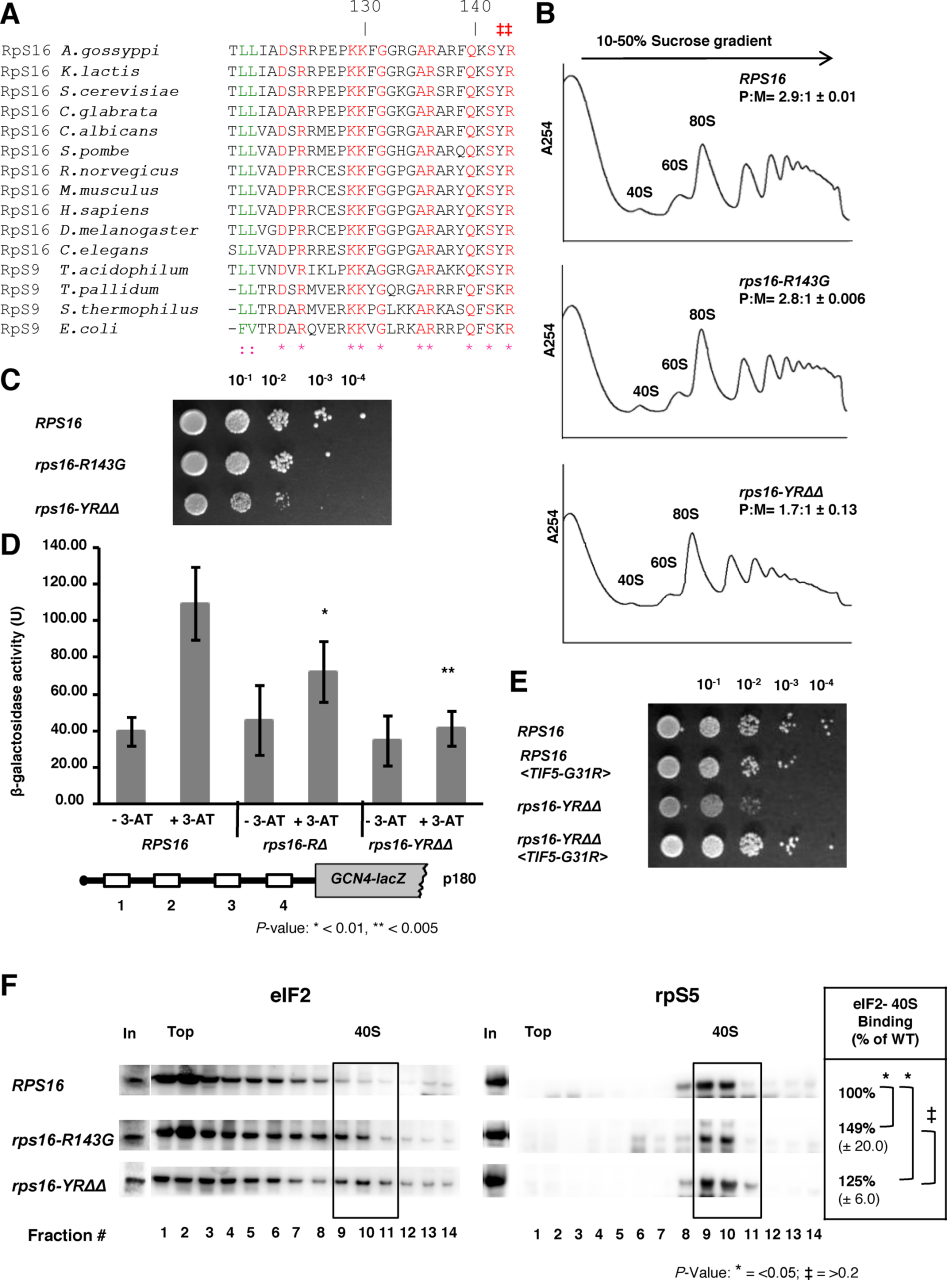
Rps16 C-terminus is important for efficient initiation. (**A**) Sequence alignments of the C-terminal region of ribosomal protein S16/S9 from various species. The last two amino acid residues (Y142 and R143 in budding yeast) which are extremely conserved are marked (‡). (**B**) Whole cell extracts from wild type and mutant yeast strains (*rps16-R143G* and *rps16-YRΔΔ* double deletion mutant) were resolved on a 10–50% sucrose density gradient. The ratio of the area under the polysomal (P) and 80S peaks are shown (P:M) with ± standard errors. (**C**) Wild type, *rps16-R143G* and *rps16-YRΔΔ* mutant yeast strains were grown in YEPD medium with 2% glucose till OD ∼0.7, serial diluted and spotted onto YEPD+2% glucose plates. (**D**) Wild type, *rps16-RΔ* and *rps16-YRΔΔ* mutants were transformed with p180 and assayed for GCN4 re-initiation efficiency using 3-AT. (**E**) *RPS16, RPS16<TIF5-G31R>*, *rps16-YRΔΔ* and *rps16-YRΔΔ <TIF5-G31R>* mutants were grown in YEPD medium containing 2% glucose till OD ∼0.5, serial diluted and spotted onto YEPD+2% glucose plates for growth assays. (**F**) Extracts from wild type, *rps16-R143G* and *rps16-YRΔΔ* double deletion mutant yeast strains after treatment with formaldehyde were resolved by velocity sedimentation on 10–30% sucrose gradients. Western blot analyses of individual fractions were performed using antibodies which recognize eIF2 and the ribosomal protein S5, respectively. ‘In’ for input - represents a 7% portion of each gradient fraction. Association of eIF2 with the 40S subunits was quantified and expressed as percentage of 40S binding normalized against Rps5.

It has been previously observed that the last two C-terminal residues of Rps16, which are highly conserved in all kingdoms of life (Figure 7A), interact with the P-site tRNA (12,42). Hence, the exact location of these residues in the PIC may be critical for efficient recruitment of the eIF2·GTP·Met-tRNA_i_^Met^ TC, or for responding properly to an AUG codon in the P site during scanning. Accordingly, we asked whether mutations in these C-terminal residues in yeast Rps16 (Y142 and R143) confer phenotypes similar to those observed in the Rps5 (Δ1-46 and K45A) mutants described above. Indeed, we found that deletion of the last two residues of Rps16 (in yeast strain *rps16-YRΔΔ*) confers a Slg^−^ phenotype (Figure 7C), a decrease in the P:M ratio (Figure 7B), and a Gcn^−^ phenotype (Figure 7D and Supplementary Figure S6AB); note that *GCN4-lacZ* expression from the *rps16* mutants was assayed using 3-AT, which blocks histidine biosynthesis, instead of SM (used for *rps5* mutants) due to differences in the strain backgrounds of the *rps16* and *rps5* mutants. Replacement of the terminal Arg residue with Gly or its deletion conferred similar but less pronounced phenotypes (Figure 7B and D). Supporting the notion that the Rps16 C-terminal residues are important for the same step in initiation impacted by mutations in the Rps5 N-terminal region, we found that introduction of *SUI5* eliminated the Slg^−^ phenotype of the *rps16-YRΔΔ* mutant (Figure 7E). We further observed accumulation of eIF2 in the 43S/48S complex in the Rps16 mutant strains (Figure 7F) similar to that observed previously in strain *rps5-Δ1-46* (10) (Figure 4C). The increase in association of eIF2 with 40S ribosomal subunits in the Rps16 mutant strains as compared to WT is statistically significant (*P*-values <0.05 in wt versus *rps16-R143G* and wt versus *rps16-YRΔΔ*), however, the difference in eIF2 association between the two mutants is not (*P*-values >0.2 in *rps16-R143G* versus *rps16-YRΔΔ*) (Figure 7F). We also observed an increased association of eIF1 along with a decreased association of eIF5 with 40S of the *rps16-YRΔΔ* mutant as compared to WT, similar to what has been observed in case of *rps5* mutants (Supplementary Figure S6CD). These data support the idea that the Rps16 C-terminus cooperates with the N-terminal segments of Rps16 and Rps5 to promote hydrolysis of eIF2-bound GTP or Pi release from eIF2-GDP+Pi on AUG recognition, to ensure efficient general initiation and proper reinitiation on *GCN4* mRNA.

## DISCUSSION

Although ribosomal proteins are not involved in the catalysis of peptide bond formation (43), they do participate in a variety of activities in the translation process, such as the recruitment of tRNAs, translation factors and specific mRNAs (44–48). However, assignment of specific functions to individual eukaryotic ribosomal proteins has been very challenging, owing in part to the extreme functional cooperation between rRNA and ribosomal proteins and between ribosomal proteins themselves (49). In addition, the majority of eukaryotic ribosomal proteins are essential for cell viability, with many playing important roles in ribosome biogenesis (15,44,50,51), which impedes their extensive mutagenesis. As a result, only a few ribosomal proteins have been assigned specific functions in eukaryotic translation (52–54).

Rps5/S7 belongs to the extremely conserved family of ribosomal proteins that are present in all domains of life (45,47); however, eukaryotic Rps5 proteins contain an additional N-terminal segment (∼50–70 amino acid residues in lengths) absent in prokaryotes (Figure 5A). While the exact function of this N-terminal region was unclear, our finding that its complete removal is lethal in yeast (10) led us to hypothesize that it facilitates ribosome activities related to the presence of eukaryotic-specific translation factors (10). In particular, we argued that the N-terminal region of Rps5 modulates eIF2-bound GTP hydrolysis upon start codon recognition by the scanning 48S complex, and/or subsequent eIF2 dissociation (10).

In this report, we provide evidence that the eukaryotic specific N-terminal region of Rps5 plays an important role in start codon recognition during translation initiation and that this function involves its interaction with the eukaryotic-specific N-terminus of Rps16. Either truncating the N-terminal 46 residues of Rps5 (*rps5-Δ1-46* mutant), or alanine substitution of Lys45 (*rps5-K45A* mutant), confers a Slg^−^ phenotype and a reduced rate of bulk translation initiation, as manifested by reduced abundance of polyribosomes relative to 80S subunits. Both mutations also impair the derepression of *GCN4* mRNA translation in response to amino acid limitation, which depends on efficient reinitiation at regulatory upstream AUGs in the *GCN4* mRNA leader. We have further found that the F46A and Y49G substitutions of Rps16 residues involved in Rps16-NTD/Rps5-NTD interactions also confer slow growth phenotypes, supporting the possibility that interaction between the N-terminal regions of Rps5 and Rps16 is critical for efficient translation initiation. Examination of a panel of *GCN4-lacZ* reporters harboring different arrangements of uORFs revealed that the strong defect in the ability of scanning PICs to bypass the AUGs of the inhibitory uORFs 2–4 could not be explained by a failure of 40S subunits to resume scanning after terminating translation by uORF1, by a reduced rate of scanning between uORF1 and uORF4, or by a failure to recognize the AUG codon at uORF1. The most likely remaining explanation is that the scanning 40S subunits are delayed in reaching the AUGs at uORFs 2–4 by the presence of 48S PICs stalled at these start codons owing to a delay in completing a step of initiation following AUG recognition. This would evoke queuing of other scanning PICs behind the stalled 48S complexes and provide ample time for these queuing PICs to load the TC, overcoming the reduction in TC abundance produced in amino acid starved cells by eIF2α phosphorylation and attendant inhibition of GDP-GTP exchange on eIF2 catalyzed by eIF2B. As a result, virtually all the PICs scanning downstream from uORF1 would be forced to reinitiate at uORFs 2,3, or 4, and fail to reinitiate at the *GCN4* AUG instead, blocking derepression of *GCN4* translation.

The conclusion that initiation is impaired in the *rps5-Δ1-46* mutant at a step following AUG recognition is supported by our previous findings that eIF2 accumulates on native 40S subunits in this strain (10), implying a failure in eIF5-stimulated GTP hydrolysis by TC in the scanning complex prior to AUG recognition, a defect in releasing Pi from eIF2·GDP at the AUG codon, or a failure to release eIF2·GDP from the 48S complex. Any of these defects are expected to block the joining of 60S subunits to assemble an 80S IC competent for elongation. Consistent with this, here we observed an accumulation in native PICs of eIF1, which is normally released on AUG recognition to allow Pi release from eIF2·GDP, and the subunit joining factor eIF5B, presumably because subunit joining cannot be completed.

Another phenotype of the *rps5-Δ1-46* and *rps5-K45A* mutants consistent with a defect following start codon recognition is a reduction in the efficiency of utilizing near-cognate UUG start codons relative to AUG codons. The U:U mismatch between UUG and the anticodon of tRNAi destabilizes the closed, P_IN_ state of the PIC from which eIF1 dissociates, Pi is released from eIF2·GDP·Pi and 60S subunit joining occurs (31). It can be expected that the delay in these reactions evoked by the U:U mismatch at UUG codons would be exacerbated by the defect postulated above engendered by alterations in the Rps5-NTD, to evoke a greater reduction in UUG versus AUG initiation in the *rps5-Δ1-46* and *K45A* mutants.

Another line of evidence supporting our model is that the Slg^−^ and Gcn^−^ phenotypes and accumulation of eIF2 on native 40S subunits observed in the *rps5-Δ1-46* mutant were all mitigated by introducing into the strain the dominant *SUI5* allele of *TIF5*, encoding the eIF5-G31R variant. In contrast to the reduced UUG:AUG initiation ratio provoked by the Rps5 mutants, *SUI5* confers an increased UUG:AUG ratio (Sui^−^ phenotype). The dominance of the Sui^−^ phenotype conferred by *SUI5* implies an alteration of eIF5 function, and previous biochemical analysis suggested that eIF5-G31R increases eIF5 GAP function, elevating the rate of GTP hydrolysis by the eIF2 TC in a model assay containing these two factors, the 40S subunit and AUG triplet. In the current model of scanning, GTP hydrolysis and establishment of an internal equilibrium between GTP and GDP·Pi occurs in the scanning PIC prior to AUG recognition, with AUG recognition triggering Pi release rather than GTP hydrolysis *per se* (31,55). If the Rps5 NTD mutations impair GTP hydrolysis in the scanning complex, then it could be proposed that introducing eIF5-G31R into these mutants restores a more nearly WT rate of GTP hydrolysis, accounting for suppression of the Rps5 mutant phenotypes by *SUI5*.

Other studies in a more fully reconstituted yeast initiation system containing eIFs -1 and -1A and a model mRNA revealed that eIF5-G31R enhances rearrangement of the PIC from an open conformation thought to be compatible with scanning, to which eIF1A and TC are bound in a relatively unstable configuration (P_OUT_), to a more closed state thought to be conducive to downstream steps of initiation, to which eIF1A and TC are bound more tightly (P_IN_) (31,39,40). In these latter studies, the ability of eIF5-G31R to promote the closed, P_IN_ conformation was restricted to PICs reconstituted with mRNAs harboring a near-cognate start codon, including UUG, which is fully consistent with the Sui^−^ phenotype of *SUI5*. As this altered activity of eIF5-G31R was observed in assays using a nonhydrolyzable GTP analog to form the eIF2 TC, they clearly occur independently of eIF5 GAP function. It seems difficult to account for the ability of *SUI5* to mitigate the effects of the Rps5 NTD mutations on initiation at AUG codons by invoking the ability of eIF5-G31R to stabilize the closed, P_IN_ state, as this activity is restricted to near-cognate codons. It is possible, however, that eIF5-G31R can stabilize the closed/P_IN_ state at AUG codons in the context of the impaired PICs formed by the Rps5 NTD variants.

Our data suggest that Rps5 and specifically its NTD is (either directly or indirectly) involved in modulating critical activities in the initiation pathway. Because the Rps5-NTD interacts with the Rps16 NTD, with a direct contact involving the residues altered by the Rps5-NTD substitution K45A (Lys-45) and Rps16-NTD F46A (Phe-46), we consider it likely that the phenotypes of the Rps5-NTD mutations involve, at least in part, disruption of the interaction between these two eukaryotic-specific segments of Rps5 and Rps16. Furthermore, the fact that Rps16 contains a long unstructured CTT that appears to make direct contact with Met-tRNAi^Met^ when the latter is base-paired with AUG in the P site (2,3,12) raises the intriguing possibility that impairing interaction between the NTDs of Rps5 and Rps16 will in turn impede productive interaction between the Rps16 CTT and Met-tRNAi^Met^ in the P site of 48S PICs, thereby accounting for mutant phenotypes of the Rps5 NTD substitutions. Supporting this idea, we found that deleting the last two residues of the Rps16 CTT confers the same phenotypes we described for the Rps5 NTD mutations, namely, a Slg^−^ phenotype, reduced rate of bulk translation initiation (decreased polysome content), impaired derepression of *GCN4* mRNA translation (Gcn^−^ phenotype) and accumulation of eIF2 on native 40S subunits. Moreover, the Slg^−^ phenotype conferred by this *rps16-YRΔΔ* allele was suppressed by *SUI5.* This concordance of genetic and biochemical phenotypes between the Rps5-NTD mutations and the *rps16-YRΔΔ* substitution strongly suggests that they arise from a common molecular lesion, which we propose to be impaired Rps16-CTT interaction with Met-tRNAi^Met^ base-paired with AUG in the P site. This defect would evoke reduced GTP hydrolysis, slower Pi release, or a delay in the conformational rearrangement from the open/P_OUT_ configuration to the closed/P_IN_ state required for steps following AUG recognition.

**Figure 8.**
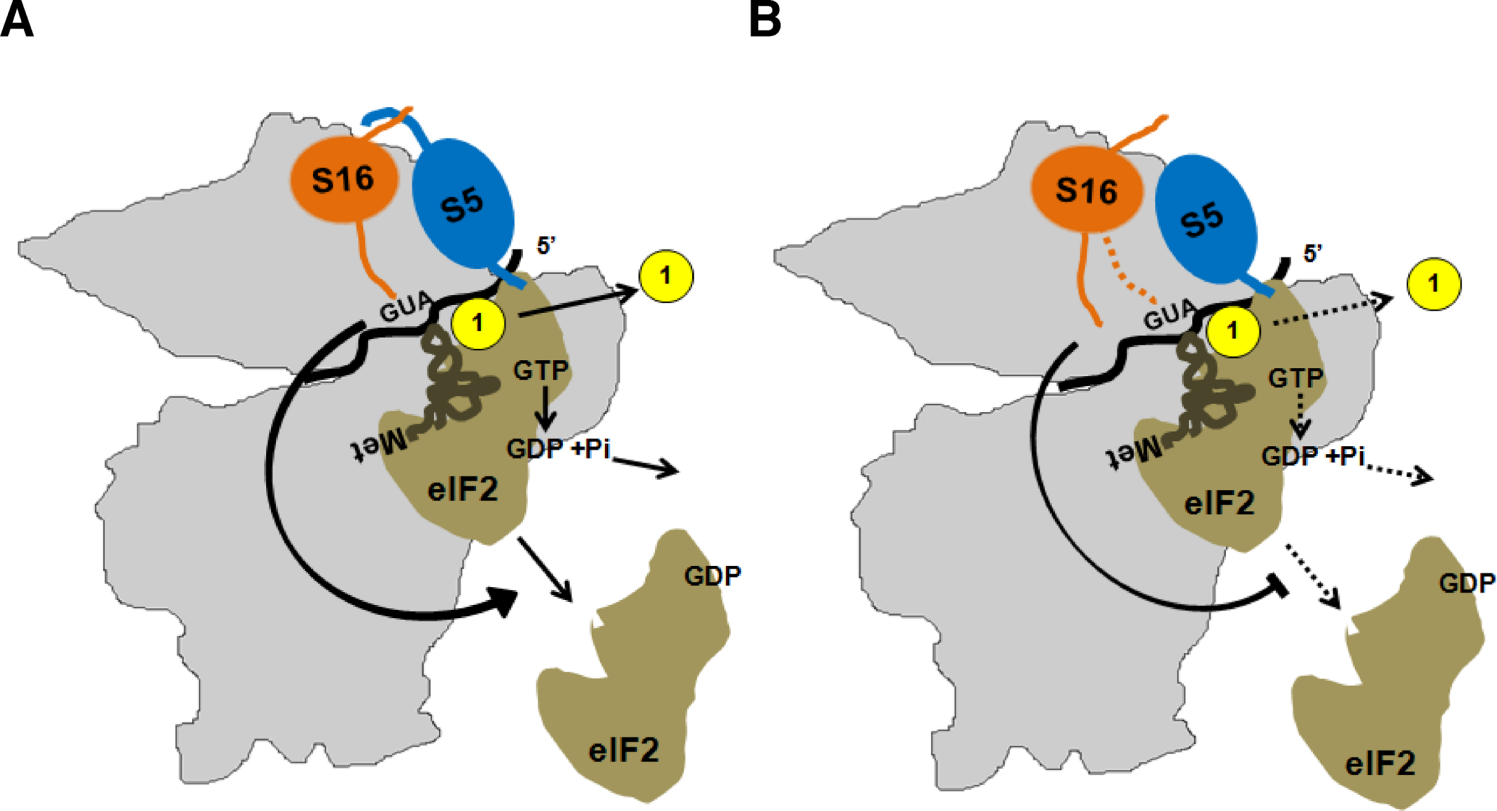
Proposed model for Rps5-Rps16 interaction affecting recruitment of eIF2·GTP·Met-tRNA_i_^Met^ ternary complex and the eIF5 stimulated hydrolysis of eIF2-bound GTP. (**A**) Rps5 N-terminal region mediates critical interaction with Rps16, whose C-terminal region extends towards the mRNA cleft and the P-site and affects the dynamic events surrounding recruitment of TC, eIF1 stimulated recognition of AUG codon, subsequent eIF5 stimulated hydrolysis of eIF2-bound GTP and the release of eIF2. (**B**) Truncation of N-terminal 46 amino acids abolishes Rps5: Rps16 interaction, and affects regulated (via Rps16 C-terminal end) recruitment of TC and eIF5 stimulated hydrolysis of eIF2-bound GTP and the release of eIF2.

There is recent evidence that domain 1 (D1) of eIF2α contacts the Rps5 C-terminal helix in mammalian PICs (2). This position of D1 is consistent with the reported interaction of eIF2α and the -3 position of the mRNA in 48S complexes (56). Moreover, it was very recently found that mammalian eIF2α crosslinks with Rps5 N-terminal (^2^TEWETAAPAVAETPDIK^18^), middle (^72^LTNSMMMHGRNNGK^85^) and C-terminal (^165^NIKTIAECLADELINAAK^182^) regions (57). It needs to be emphasized, however, that while the middle and C-terminal regions are extremely conserved among all Rps5 homologs, the N-terminal region mentioned above is rather dissimilar between yeast and mammalian proteins (Figure 5A). It thus remains possible that the mechanism of eIF2 recruitment in mammals and yeast differs slightly and/or that the Rps5 C-terminal region is primarily responsible for eIF2-Rps5 interaction (consistent with the study of Hashem *et al*. (2)) and the Rps5 N-terminal and middle regions only fine-tune the interaction. We thus hypothesize that interaction between eIF2 and Rps5 and subsequent Rps5-Rps16 association modulates the location of the Rps16 CTT to promote correct placement of TC in the P site and eIF5-stimulated GTP-hydrolysis or Pi release (Figure 8). The interaction between Rps5 and Rps16 may also indirectly influence the placement of eIF1, TC and eIF5 following start codon recognition. It is important to mention a very recent report indicating that positioning of the C-terminal tail of rpS9 (bacterial counterpart of Rps16) is important for fidelity of translation initiation in bacteria (58); although a molecular understanding of this effect is still lacking. Our study, however, provides some of the first evidence supporting the functional significance of protein–protein interactions within the ribosome that are absent in prokaryotes but represent a defining feature of eukaryotic ribosomes.

## SUPPLEMENTARY DATA

Supplementary Data are available at NAR Online.

SUPPLEMENTARY DATA
