# Supplementary material for: Rps5-Rps16 communication is essential for efficient translation initiation in yeast S. cerevisiae
Source: Nucleic Acids Res. 2014 Jun 21;42(13):8537–55. doi: 10.1093/nar/gku550 (PMC4117775; doi:10.1093/nar/gku550)
Supplement: SUPPLEMENTARY DATA [file supp_42_13_8537__index.html]

Rps5-Rps16 communication is essential for efficient translation initiation in yeast S. cerevisiae — SUPPLEMENTARY DATA 

# Rps5-Rps16 communication is essential for efficient translation initiation in yeast *S. cerevisiae*

## SUPPLEMENTARY DATA

**Files in this Data Supplement:**

- SUPPLEMENTARY DATA
- SUPPLEMENTARY DATA
