# Supplementary material for: Rps5-Rps16 communication is essential for efficient translation initiation in yeast S. cerevisiae
Source: Nucleic Acids Res. 2014 Jun 21;42(13):8537–55. doi: 10.1093/nar/gku550 (PMC4117775; doi:10.1093/nar/gku550)
Supplement: SUPPLEMENTARY DATA [file supp_gku550_nar-02957-a-2013-File004.pdf]

Ghosh_Fig S1

A

Strain / Construct	***rps5-Δ0***		***rps5-Δ01-13***		***rps5-Δ1-24***		***rps5-Δ1-30***		***rps5-Δ1-46***	
	**-SM**	**+SM**	**-SM**	**+SM**	**-SM**	**+SM**	**-SM**	**+SM**	**-SM**	**+SM**
**p180**	**13.99** (±0.97)	**38.39** (±3.21)	**13.58** (±1.06)	**31.40** (±3.25)	**13.42** (±1.36)	**30.57** (±5.77)	**3.01** (±0.26)	**4.66** (±0.52)	**0.24** (±0.04)	**0.27** * (±0.10)
**p196**	**16.59** (±1.47)	**34.23** (±5.27)	**13.14** (±1.48)	**25.46** (±2.29)	**13.55** (±0.80)	**33.30** (±2.03)	**2.87** (±0.27)	**4.68** (±0.42)	**1.51** (±0.16)	**1.94** * (±0.31)
**p227**	**161.63** (±9.19)	**144.31** (±23.71)	**150.51** (±9.90)	**143.88** (±19.82)	**146.25** (±19.50)	**137.94** (±21.11)	**92.83** (±8.37)	**88.60** (±10.98)	**90.04** (±3.91)	**74.36** (±3.62)
**p209**	**149.74** (±26.63)	**167.24** (±40.53)	**121.51** (±7.62)	**131.34** (±10.03)	**170.73** (±10.25)	**171.55** (±21.01)	**160.73** (±30.95)	**186.22** (±39.58)	**46.33** (±8.53)	**47.23** (±14.09)
**p226**	**3.31** (±0.40)	**4.31** (±0.54)	**2.11** (±0.41)	**2.72** (±0.45)	**1.51** (±0.09)	**1.72** (±0.12)	**1.32** (±0.10)	**1.51** (±0.11)	**3.52** (±0.47)	**4.70** (±0.35)
**pM226**	**2.09** (±0.48)	**2.43** (±0.38)	**2.74** (±0.55)	**2.36** (±0.20)	**2.53** (±0.49)	**2.46** (±0.57)	**1.99** (±0.20)	**2.47** (±0.49)	**3.99** (±0.56)	**3.43** (±0.51)

P -value: * = <0.001

B

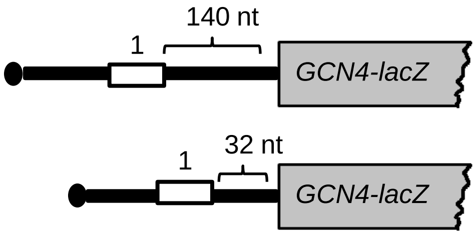

**Construct**	**β-gal activity (U)**		
	**SM**	***rps5-Δ0***	***rps5-Δ1-46***
**pM199**	**-**	**68.48** (±4.17)	**41.56** (±4.93)
**pG67**	**-**	**15.08** (±1.48)	**6.72** (±1.21)

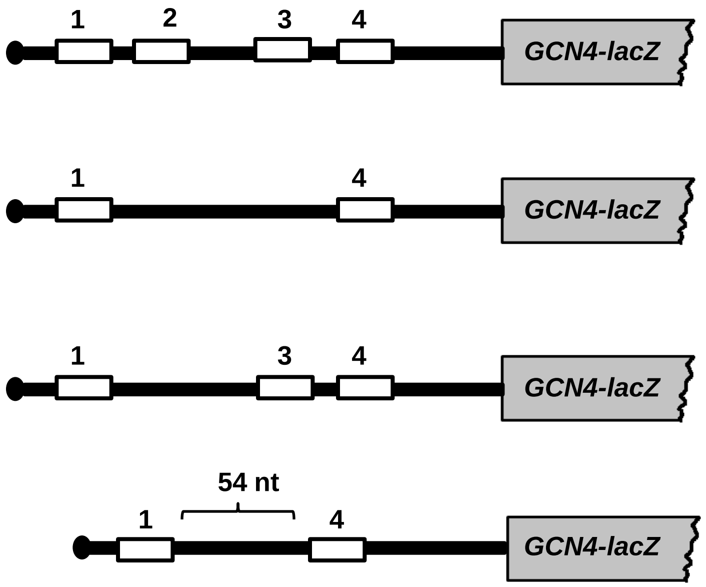

Construct	β-gal activity (U)		
	SM	*rps5-Δ0*	*rps5-Δ1-46*
**p180**	-	**5.66** (±1.15)	**0.97** (±0.25)
	+	**15.92** (±1.61)	**1.10** * (±0.24)
**p196**	-	**4.85** (±1.01)	**1.48** (±0.37)
	+	**15.52** (±2.28)	**2.01** * (±0.26)
**p195**	-	**4.21** (±1.25)	**1.90** (±0.24)
	+	**14.77** (±1.55)	**1.64** (±0.24)
**pG29**	-	**9.89** (±1.07)	**5.35** (±1.38)
	+	**12.05** (±2.15)	**2.98** (±0.21)

P -value: * = <0.001

β-galactosidase activity (U)

	AUG	UUG	UUG/AUG
rps5-Δ0	**9.40**	**0.21**	**0.023**
rps5-Δ1-46	**3.04**	**0.02**	**0.005**
rps5-K45A	**26.93**	**0.37**	**0.014**

A

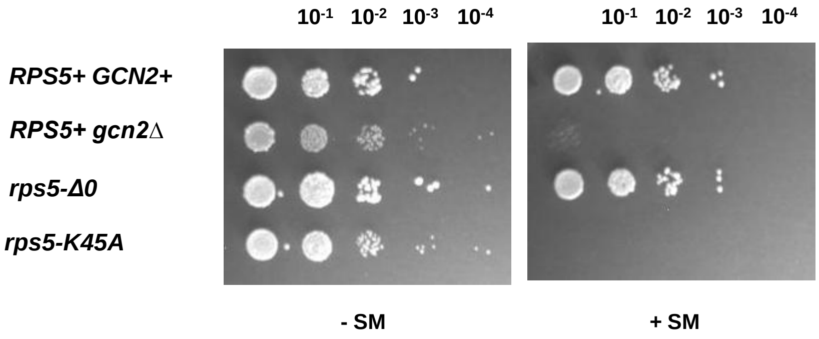

B

Strain / Construct	*rps5-Δ0*		*rps5-K45A*	
	-SM	+SM	-SM	+SM
p180	**11.52** (±1.28)	**20.52** (±3.58)	**6.27** (±1.27)	**8.95** (±0.97)

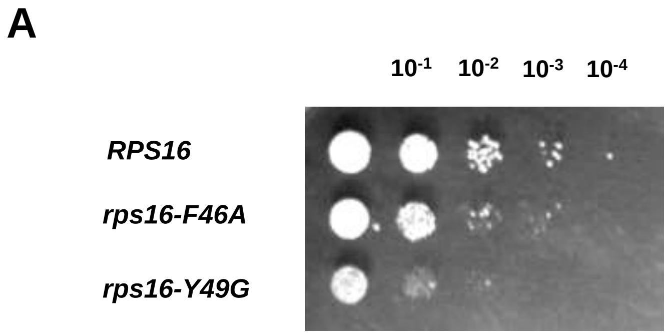
A
10-1 10-2 10-3 10-4
RPS16
rps16-F46A
rps16-Y49G

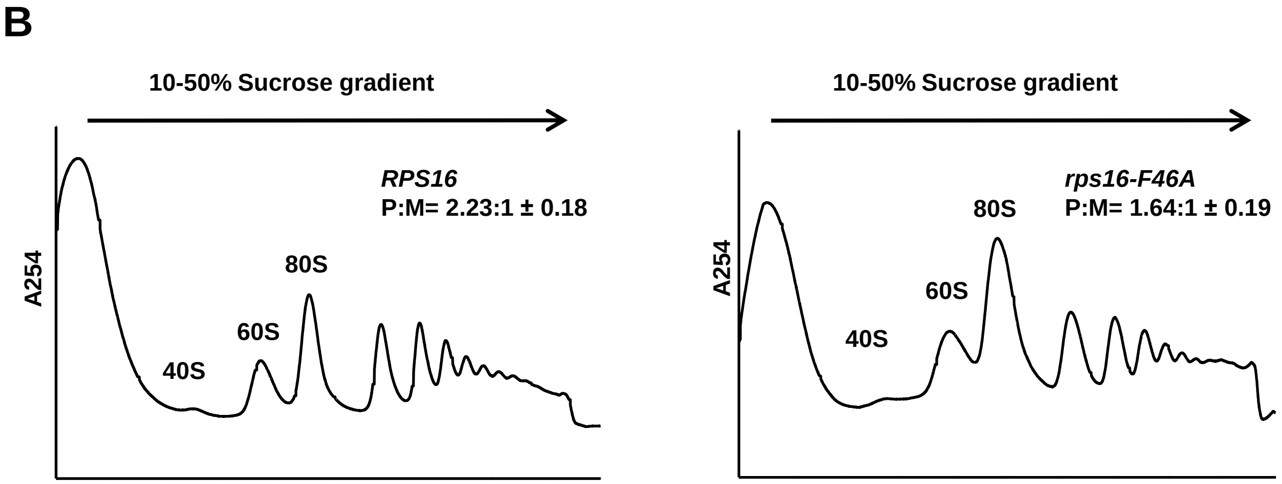
B
10-50% Sucrose gradient
RPS16
P:M= 2.23:1 ± 0.18
A254
80S
60S
40S
10-50% Sucrose gradient
rps16-F46A
P:M= 1.64:1 ± 0.19
A254
80S
60S
40S

Ghosh_Fig S6

A

Construct \ Strains	*RPS16*		*rps16-RΔ*		*rps16-YRΔΔ*	
	- 3-AT	+ 3-AT	- 3-AT	+ 3-AT	- 3-AT	+ 3-AT
p180	**39.64** (±7.63)	**109.24** (±19.57)	**45.70** (±18.96)	**72.11** (±16.41)	**34.44** (±13.82)	**41.03** (±9.58)

B

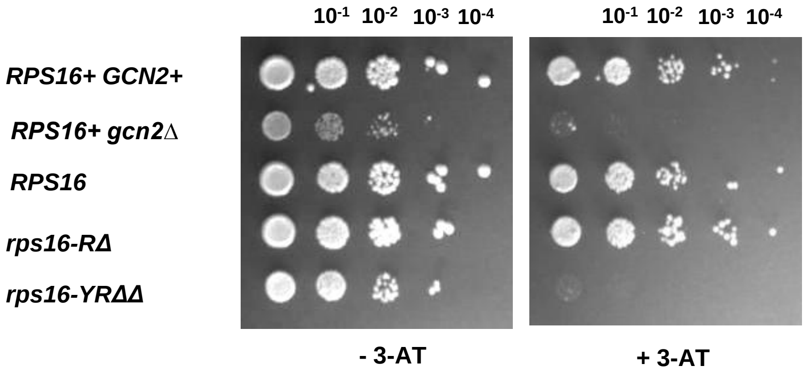

C

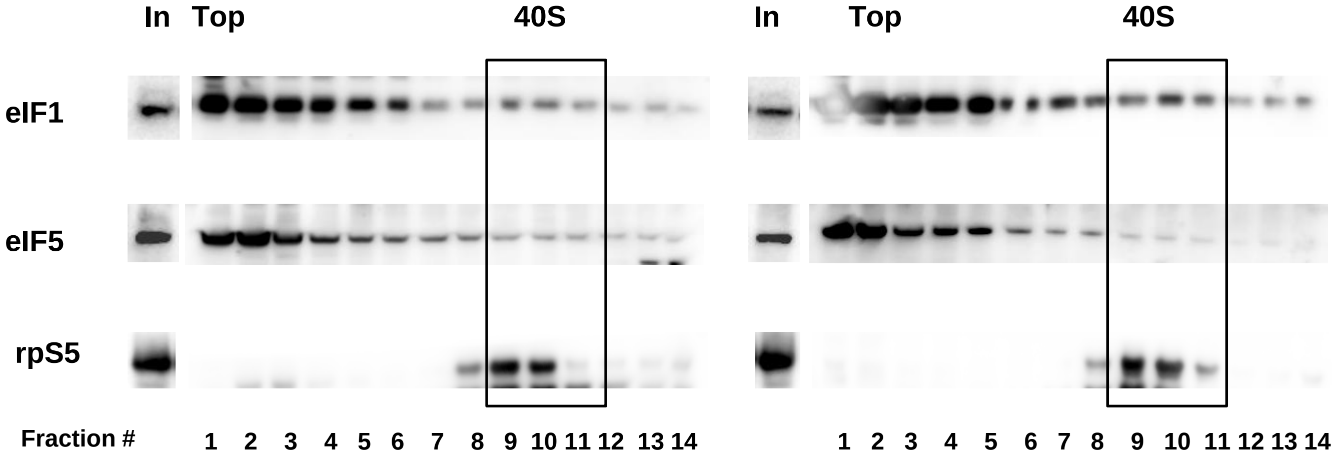

D

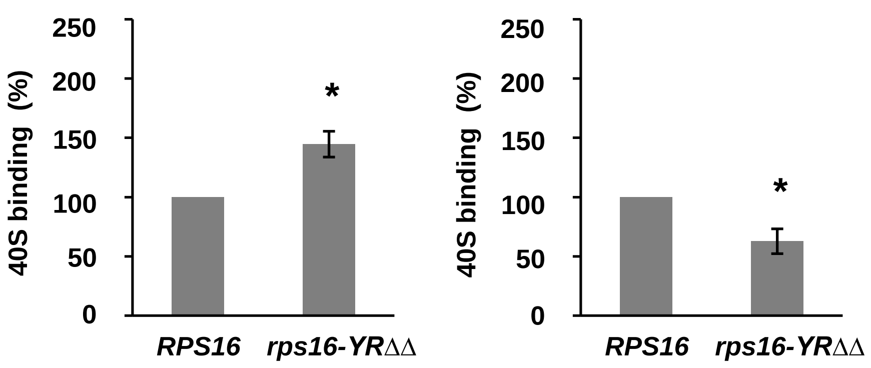

P-Value: * = <0.005
