# Supplementary material for: Rps5-Rps16 communication is essential for efficient translation initiation in yeast S. cerevisiae
Source: Nucleic Acids Res. 2014 Jun 21;42(13):8537–55. doi: 10.1093/nar/gku550 (PMC4117775; doi:10.1093/nar/gku550)
Supplement: SUPPLEMENTARY DATA [file supp_gku550_nar-02957-a-2013-File003.doc]

**SUPPLEMENTAL MATERIAL**

**FIGURE LEGENDS**

**FIGURE S1. Expression of reporter *GCN4-lacZ* constructs in WT and mutant yeast strains**.  **(A)** β-Galactosidase activity (units)/raw values as presented in Figs. 1 and 2 measured under normal (-SM) and amino acids starved conditions (+SM) are shown. The standard errors are shown in parentheses. P-values were calculated for *rps5-∆1-46* strains under amino acids starved conditions (+SM) to compare the statistical significance of the increased expression from p196 with respect to p180. **(B)** Expression of the reporter pM199 and pG67 constructs (containing only uORF1) in *rps5-∆0* and *rps5-∆1-46* strains under normal (-SM) conditions; β-galactosidase activity (units)/raw values are shown. The standard errors are shown in parentheses.

**FIGURE S2. Expression of reporter *GCN4-lacZ* constructs in *rps5-∆0* and *rps5-∆1-46* strains.** β-Galactosidase activity (units)/raw values measured under normal (-SM) and amino acids starved conditions (+SM) are shown. The standard errors are shown in parentheses. P-values were calculated for *rps5-∆1-46* strains under amino acids starved conditions (+SM) to compare the statistical significance of the increased expression from p196 with respect to p180.

**FIGURE S3. Expression of reporter *HIS4-lacZ* constructs in *rps5-∆0* and *rps5-∆1-46* and *rps5-K45A strains.*** β-Galactosidase activity (units)/raw values are shown along with UUG/AUG ratios as presented in Fig. 3C.

**FIGURE S4. Gcn phenotypes of the WT and *rps5-K45A* mutant yeast strains.** **(A)** Yeast cell growth. Serial dilutions of strains spotted onto minimal media under non-starved (-SM), or amino acid (aa) starved conditions (+SM), respectively.

**(B)** Expression of the reporter p180 construct in *rps5-∆0* and *rps5-K45A* yeast strains under non-starved (-SM), or amino acid (aa) starved conditions (+SM), respectively. β-Galactosidase activity (units)/raw values as presented in Fig. 6C are shown.

**FIGURE S5. Single point F46A and Y49G mutations in Rps16 confer slow growth phenotypes. (A)** Yeast cell growth, serial dilutions. **(B)** Ribosome profiles of the WT, *rps16* and *rps16-F46A* mutant yeast strains. Extracts were resolved in 10-50% sucrose density gradients. The ratios of the area under the polysomal (P) and 80S (M) peaks (P:M) are shown with +/- standard errors.

**FIGURE S6. Gcn phenotypes and association of eIF1 and eIF5 with the 40S ribosomal subunit of WT and *rps16* mutant yeast strains.** **(A)** Expression of the reporter p180 construct in WT, *rps16-R∆* and *rps16-YR∆∆* yeast strains under non-starved (-3-AT), or amino acid (aa) starved conditions (+3-AT), respectively. β-Galactosidase activity (units)/raw values as presented in Fig. 7D are shown. **(B)** Yeast cell growth. Serial dilutions of strains spotted onto minimal media under non-starved (-3-AT), or amino acid (aa) starved conditions (+3-AT), respectively. **(C)** Extracts from isogenic wt and Rps16 mutant strains were resolved by sucrose density gradient (10-30%) sedimentation. Western blot analyses were done using antibodies against eIF1, eIF5 and the ribosomal protein S5, respectively. “In” for input - represents a 7% portion of each gradient fraction. Analysis of eIF1 and eIF5 association was done using whole cell extract cross-linking with formaldehyde. **(D)** Association of eIF1 and 5 with the 40S was quantified and expressed as % of 40S binding normalized against Rps5.

**MATERIALS AND METHODS**

**β-Galactosidase assays**

Reporter plasmids p180, p196, p195, pG29, pM199 and pG67 has been described previously (1,2). For *GCN4-lacZ* assays, cells were grown in a minimal synthetic (SD) medium supplemented with appropriate amino acids containing 2% galactose (for 2 h). To invoke amino acid starvation, sulfometuron methyl was then added to a final concentration of 1 µg/ml, and the incubation was continued for additional 5 h. Cells were harvested, and extracts were prepared by subsequent cycles of cell freezing in liquid nitrogen and thawing at 37°C. β-Galactosidase activity was measured following the protocol described in Clontech Yeast Protocols Handbook using *O*-nitrophenyl β-D-galactopyranoside as a substrate. For yeast growth assays, serial dilutions of strains were spotted onto minimal media under non-starved (-SM or – 3-AT), or amino acid (aa) starved conditions (+SM or + 3-AT) containing Sulfometuron methyl (1 µg/ml) or 3-AT (5 mM).

**Polyribosome analysis**

Fractionation of polyribosomes was done essentially as described before (3,4) using 10-50% (17000 rpm., 18 h) sucrose gradients and a Beckman SW32.1 rotor. All procedures were performed at 4°C. Yeast cells from 50 ml of log phase culture were pelleted, treated for 10 min with 100 µg/ml cycloheximide and repelleted. Cell extracts were made by glass bead cell disruption (3-5 cycles of 1 min each), with intermittent cooling on ice. The following buffer was used: 100 mM KCl, 2.5 mM magnesium acetate, 20 mM HEPES•KOH, pH 7.4, 14.4 mM β-mercaptoethanol, 100 µg/ml cycloheximide. Cell debris was removed by centrifugation at 7000 rpm for 8 min and polyribosomes were resolved by sucrose density gradient centrifugation as indicated. Gradients were collected using the ISCO Programmable Density Gradient System with continuous monitoring at 254 nm using an ISCO UA-6 absorbance detector. Analysis of ratios of 80S monosomes to polyribosomes was done essentially as before (4). Fractionation of cell extracts using formaldehyde cross-linking was done as described by Nielsen and co-authors (2).

**REFERENCES**

1. Grant,C.M., Miller,P.F. and Hinnebusch,A.G. (1994) Requirements for intercistronic distance and level of eIF-2 activity in reinitiation on GCN4 mRNA varies with the downstream cistron. *Mol. Cell. Biol.,* **14,** 2616-2628.

2. Nielsen,K.H., Szamecz,B., Valášek,L., Jivotovskaya,A., Shin,B.S. and Hinnebusch,A.G. (2004) Functions of eIF3 downstream of 48S assembly impact AUG recognition and GCN4 translational control. *EMBO,* **23,** 1166-1177.

3. Galkin,O., Bentley,A.A., Gupta,S., Compton,B.A., Mazumder,B., Kinzy,T.G., Merrick,W.C., Hatzoglou,M., Pestova,T.V., Hellen,C.U. and Komar,A.A. (2007) Roles of the negatively charged N-terminal extension of Saccharomyces cerevisiae ribosomal protein S5 revealed by characterization of a yeast strain containing human ribosomal protein S5. *RNA,* **13,** 2116–2128.

4. Lumsden,T., Bentley,A.A., Beutler,W., Ghosh,A., Galkin,O. and Komar,A.A. (2010) Yeast strains with N-terminally truncated ribosomal protein S5: implications for the evolution, structure and function of the Rps5/RpS7 proteins. *Nucleic Acids Res.,* **38,** 1261-1272.
